# Transient non-specific DNA binding dominates the target search of bacterial DNA-binding proteins

**DOI:** 10.1016/j.molcel.2021.01.039

**Published:** 2021-04-01

**Authors:** Mathew Stracy, Jakob Schweizer, David J. Sherratt, Achillefs N. Kapanidis, Stephan Uphoff, Christian Lesterlin

**Affiliations:** 1 Department of Biochemistry, University of Oxford, Oxford OX1 3QU, UK; 2 Max Planck Institute for Dynamics of Complex Technical Systems, 39106 Magdeburg, Germany; 3 Biological Physics Research Group, Clarendon Laboratory, Department of Physics, University of Oxford, Oxford OX1 3PU, UK; 4 Molecular Microbiology and Structural Biochemistry (MMSB), Université Lyon 1, CNRS, INSERM, UMR5086, 69007 Lyon, France

**Keywords:** target search of bacterial DNA-binding proteins, single-molecule tracking, chromosome-free cells, Chromosome-crowding

## Abstract

Despite their diverse biochemical characteristics and functions, all DNA-binding proteins share the ability to accurately locate their target sites among the vast excess of non-target DNA. Toward identifying universal mechanisms of the target search, we used single-molecule tracking of 11 diverse DNA-binding proteins in living *Escherichia coli*. The mobility of these proteins during the target search was dictated by DNA interactions rather than by their molecular weights. By generating cells devoid of all chromosomal DNA, we discovered that the nucleoid is not a physical barrier for protein diffusion but significantly slows the motion of DNA-binding proteins through frequent short-lived DNA interactions. The representative DNA-binding proteins (irrespective of their size, concentration, or function) spend the majority (58%–99%) of their search time bound to DNA and occupy as much as ∼30% of the chromosomal DNA at any time. Chromosome crowding likely has important implications for the function of all DNA-binding proteins.

## Highlights

• Protein motion was compared between unperturbed cells and DNA-free cells
• Protein mobility was dictated by DNA interactions rather than molecular weight
• The nucleoid is not a physical barrier for protein diffusion
• The proteins studied spend most (58%–99%) of their search time bound to DNA

## Graphical abstract

**Figure:**
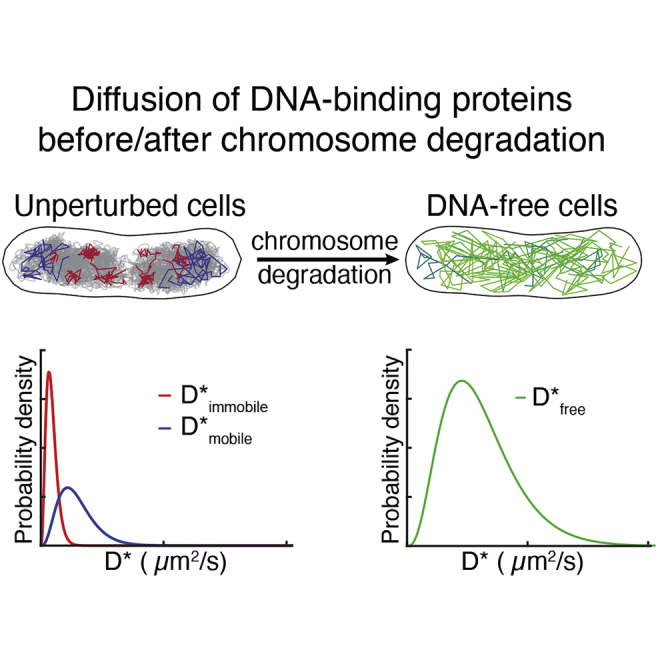

## Teaser

To understand how DNA-binding proteins find their target sites, Stracy et al. tracked the motion of 11 diverse proteins in living *Escherichia coli*. By comparing protein behavior in normal cells and cells without chromosomes, they showed that the DNA-binding proteins spend most of their search time bound to DNA.

## Introduction

DNA is organized into chromosomes that must be maintained in a highly compacted state while keeping the genetic information accessible for processing by many DNA-binding proteins. The ability of these proteins to identify and bind to specific DNA target sites among the vast excess of non-target DNA is crucial for many fundamental cellular functions, including recruitment of transcription factors to promoter sequences, of DNA repair proteins to DNA lesions, or of DNA topoisomerases to supercoiled DNA strands. In all organisms, diffusion is the primary mechanism by which DNA-binding proteins locate their targets (Erbaş and Marko, 2019; Erbaş et al., 2019; Schavemaker et al., 2018). The diffusion coefficient of a particle in a dilute solution is determined by its size and the viscosity and temperature of the medium. In the crowded and heterogeneous intracellular environment, however, a myriad of specific and non-specific interactions and steric effects influence the mobility of macromolecules. Because of this complexity, efforts to understand molecular mobility have relied on phenomenological models (Kalwarczyk et al., 2012; Mika and Poolman, 2011) or coarse-grained simulations of the cytoplasm (Chow and Skolnick, 2017; Feig et al., 2015; Hasnain et al., 2014). In this context, analysis of *in vivo* experimental data is crucial not only to determine parameter values but also the structure of such models by informing which cellular components and interactions should be included in a model.

Contrary to eukaryotes, bacterial chromosomes are not compartmentalized into a nucleus but organized and compacted into nucleoid structures without a physical barrier from the cytoplasm. A long-standing question is whether the presence of the dense nucleoid mesh affects the mobility of all cytoplasmic proteins, regardless of their ability to bind DNA, because it could pose a steric barrier preventing larger proteins from accessing the densest regions of the nucleoid (Kalwarczyk et al., 2012; Konopka et al., 2006; Kuznetsova et al., 2014). Furthermore, the target search process is subject to a trade-off between speed and accuracy to distinguish target from non-target sites (Zandarashvili et al., 2015). Accumulating experimental evidence supports theoretical considerations that the search efficiency is maximized by “facilitated diffusion”—the combination of 3D protein diffusion with non-specific binding and 1D sliding along DNA (Halford and Marko, 2004; Hammar et al., 2012; von Hippel and Berg, 1989). Together with chromosome crowding effects, the relative contribution of 3D and 1D diffusion modes during the target search should strongly affect the overall mobility of DNA-binding proteins *in vivo*.

Ensemble fluorescence methods have been used to investigate protein mobility in live bacterial cells (Bacia et al., 2006; Cluzel et al., 2000; Konopka et al., 2006; Kumar et al., 2010; Mika and Poolman, 2011; Mika et al., 2010; Mullineaux et al., 2006; Nenninger et al., 2010; Ramadurai et al., 2009). More recently, it has also become possible to directly visualize aspects of the target search of individual proteins in live cells using single-molecule microscopy (Elf et al., 2007; Hammar et al., 2012; Kapanidis et al., 2018; Normanno et al., 2015; Rhodes et al., 2017). These studies focused on a limited number of test proteins, typically transcription factors, raising the question of whether the proposed models for the target search are universal for diverse types of DNA-binding proteins. Although the observed intracellular mobility and spatial distribution of DNA-binding proteins suggest that non-specific DNA binding contributes to the target search, these interactions appeared too transiently for direct visualization and quantification by live-cell imaging (Garza de Leon et al., 2017; Stracy et al., 2015, 2016; Uphoff et al., 2013). In previous attempts to resolve this issue, the DNA-binding affinity of the studied protein was perturbed genetically (Elf et al., 2007), but for some proteins, this is not possible. Alternatively, protein mobility has been compared between different regions of the cell with lower or higher DNA density (Bakshi et al., 2011; Sanamrad et al., 2014; Stracy et al., 2015). However, because few DNA-binding proteins are located in DNA-free regions of the cell, it is difficult to accurately measure their diffusion with this approach (Le Gall et al., 2017; Stracy et al., 2015).

To overcome this uncertainty and determine the influence of the nucleoid DNA on protein mobility, we compared protein mobility in unperturbed cells and cells engineered to degrade all of their chromosomal DNA. Combining this approach with diffusion simulations allowed us to quantitatively partition the behavior of 11 diverse DNA-binding proteins into long-lived DNA-binding at target sites, transient non-specific DNA-binding, and free diffusion between DNA strands. We found that the representative DNA-binding proteins (irrespective of their size, concentration, or function) spend the majority of their search time bound to DNA, occupying as much as ∼30% of the chromosomal DNA at any time.

## Results

### Live-cell, single-molecule tracking of a variety of DNA-binding proteins

To uncover universal mechanisms that govern the target search process of DNA-binding proteins, we measured the diffusion characteristics of 11 proteins involved in various DNA transactions and spanning a large range of molecular weights and intracellular concentrations. We included proteins whose target is a specific DNA sequence, such as RNA polymerase (RNAP), the low-copy-number transcription factor LacI, and the abundant histone-like, nucleoid-associated proteins HU and H-NS. We further analyzed proteins that target DNA structural motifs, such as topoisomerases (TopoIV and Gyrase) acting on supercoiled DNA, or DNA polymerase I (Pol1) and DNA ligase (LigA), which recognize gapped or nicked DNA, respectively. We also studied DNA repair proteins recognizing DNA lesions (UvrA) or mismatches (MutS) and the structural maintenance of chromosomes (SMC) protein MukB, which functions in chromosome organization but binds DNA with little known specificity (Table S1).

**Figure 1:**
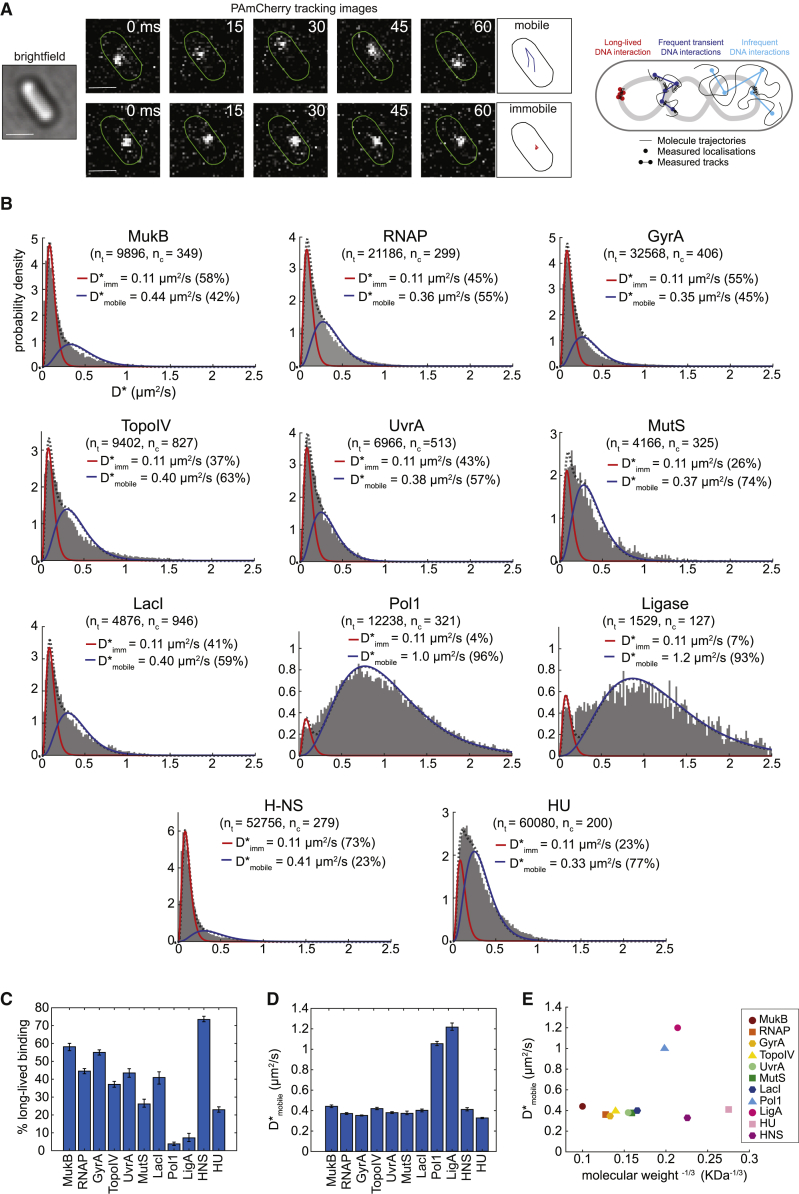
Intracellular mobility of diverse types of DNA-binding proteins in live *E. coli* cells is highly variable and unrelated to their molecular weight (A) Illustration of photoactivated single-molecule tracking, showing example fluorescence images and trajectories of a mobile and an immobile molecule. Scale bar, 1 μm. The measured localizations reflect the average position of a molecule during a frame exposure. Apparent diffusion coefficients (D^∗^) report the frequency and duration of DNA interactions by measuring the average displacements over the course of a trajectory. (B) D^∗^ histograms for diverse DNA-binding proteins, fitted with a two-species model (black dashed line) of a mixture of immobile (red) and mobile molecules (blue). The number of cells (n_c_) and the numbers of tracks (n_t_) are indicated. (C) Percentages of immobile molecules obtained from fitting the D^∗^ histograms in (B) with a two-species model. Error bars represent 95% CI. (D) D_mobile_^∗^ values for the mobile molecule populations. (E) D_mobile_^∗^ plotted against the cubic root of the molecular weight of the protein complex. See also Figure S1.

To measure protein mobility, we used single-molecule tracking (Elf et al., 2007; Gahlmann and Moerner, 2014; Li et al., 2018; Uphoff and Sherratt, 2017), using fusions to the photoactivatable fluorescent protein PAmCherry (Subach et al., 2009) that were expressed at native levels from their endogenous chromosome locus in *Escherichia coli* cells. To allow tracking of single molecules, we recorded videos with more than 5,000 frames at 15 ms/frame using sparse photoactivation so that ∼1 molecule per cell was photoactivated at any time (Bakshi et al., 2011; English et al., 2011; Manley et al., 2008; Niu and Yu, 2008; Uphoff et al., 2013). Following automated localization and particle tracking analysis, the apparent diffusion coefficient D^∗^ was calculated from the mean-squared displacement (MSD) of each trajectory (Uphoff, 2016; Figure 1A). This was done by truncating trajectories to a fixed length of 5 frames (75 ms), and we obtained D^∗^ by fitting an analytical expression to the distribution of diffusion coefficients. We reasoned that each DNA-binding protein should exist in at least two diffusive states: mobile proteins searching for target sites and proteins specifically bound to DNA, which appear to be essentially immobile because of the slow and constrained motion of chromosomal DNA (Figure 1A; Elmore et al., 2005).

### The mobility of DNA-binding proteins is independent of their molecular weight

The average intracellular mobility of the different DNA-binding proteins varied strongly, ranging from mostly immobile proteins, such as HNS (mean D^∗^ = 0.17 μm^2^/s), to mostly diffusing proteins, such as LigA (mean D^∗^ = 1.14 μm^2^/s) (Figure 1B). There was no obvious relation between the observed mobility and the type of DNA interactions (e.g., sequence-specific, structure-specific, or lesion-binding). To distinguish between proteins specifically bound to DNA and mobile proteins searching for target sites, we analyzed the motion of Pol1 molecules that become recruited to DNA damage sites (Figure S1A). DNA damage treatment increased the proportion of molecules that are immobile (with D_imm_^∗^ = 0.11 μm^2^/s) for the entire duration of a trajectory (75 ms), consistent with ∼2-s average residence of Pol1 at lesions (Uphoff et al., 2013). For other proteins in this study, we have previously observed a similar increase in “long-lived” immobile molecules (D_imm_^∗^ = 0.11 μm^2^/s) upon recruitment to specific target sites after induction of DNA damage (LigA, UvrA, and MutS; Stracy et al., 2016; Uphoff et al., 2013, 2016) or by capturing DNA-bound enzymes during catalysis by drug treatment (Gyrase and TopoIV; Stracy et al., 2019; Zawadzki et al., 2015). We previously observed a decrease in the long-lived immobile population of RNA polymerase (RNAP) upon addition of a transcription-inhibiting drug (Stracy et al., 2015) and a similar decrease for LacI after removing its chromosomal binding site (Garza de Leon et al., 2017). These studies show that the long-lived immobile population represents proteins specifically bound at DNA target sites. The apparent mobility of these DNA-bound molecules was slightly above the localization uncertainty of σ = 35 nm measured in chemically fixed cells (giving an apparent D_fixed_^∗^ = 0.07 μm^2^/s; Figure S1B). By subtracting the contribution of the localization uncertainty from the observed D^∗^ (Michalet and Berglund, 2012), we estimate the D^∗^ of proteins bound to DNA for the entire duration of a trajectory as D^∗^_bound_ = 0.04 μm^2^/s.

To determine the relative abundances and average diffusion coefficients of mobile molecules searching for target sites and long-lived immobile molecules bound to DNA, we fitted the D^∗^ histograms using an analytical function derived from a two-species Brownian motion model (Stracy et al., 2015; Figures 1B, S1A, and S1B). The quantification confirmed our initial observations that the different DNA-binding proteins exhibit vastly different mobility in cells in terms of the percentage of molecules that were mobile (ranging from 96% for Pol1 to 23% for H-NS) (Figure 1C) and in terms of the diffusion coefficients of the mobile molecules (ranging from 0.33 μm^2^/s for HU to 1.2 μm^2^/s for LigA) (Figure 1D).

According to the Stokes-Einstein equation for Brownian motion, the diffusion coefficient of a spherical particle is related to its mass: D ∼M^−1/3^. To test this relation for the DNA-binding proteins, we plotted D^∗^ of molecules in the mobile state against the known molecular weights of each protein (Figure 1E). The *in vivo* mobility of DNA-binding proteins was largely independent of their mass. Although non-spherical proteins are expected to deviate from the Stokes-Einstein law, this does not explain the absence of any correlation between mass and mobility. In contrast, previous studies showed a clear dependence of mass on the mobility of cytoplasmic proteins with no affinity for DNA (Kalwarczyk et al., 2012; Kumar et al., 2010; Nenninger et al., 2010). Our results indicate that the apparent mobility of DNA-binding proteins is dictated by molecular interactions independent of protein mass. Although it has been shown that DNA sliding may increase the efficiency of the target search for proteins that are present at low concentration in cells, theoretical and *in vitro* studies have suggested that this may not be the case for highly abundant proteins (Redding and Greene, 2013). However, we found no correlation between the mobility and the intracellular concentration of the different proteins (Figure S1C).

### DNA-binding proteins remain closely associated with the nucleoid during their target search

**Figure 2:**
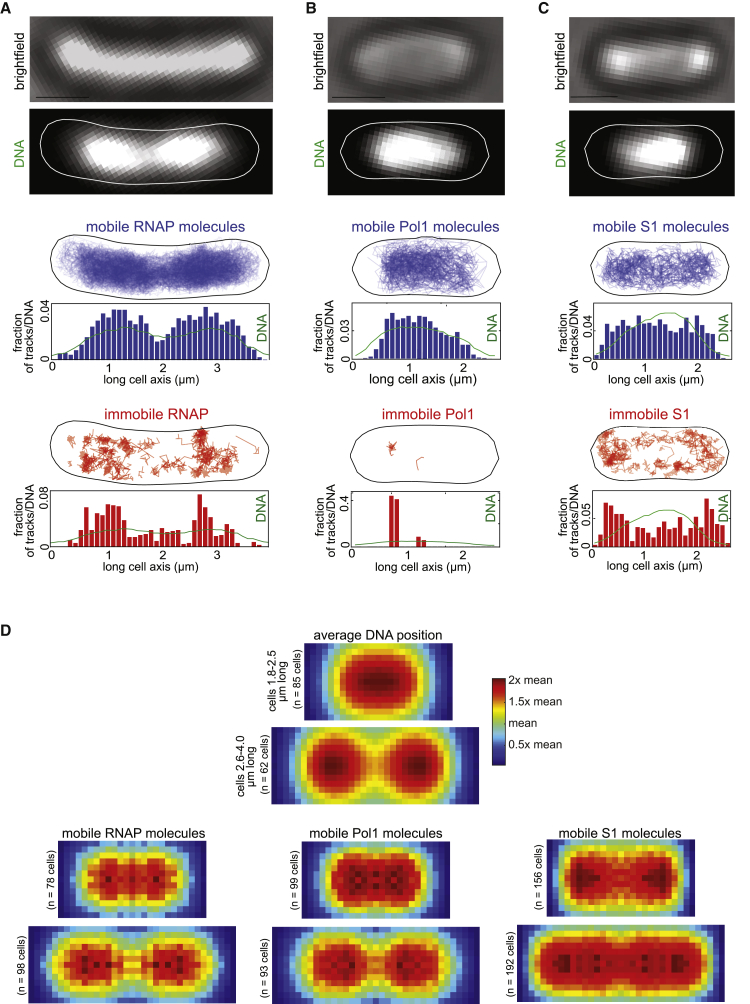
DNA-binding proteins stay closely associated with the nucleoid during the target search (A–C) Localizations of Pol1 (PolA-PAmCherry), (B) RNAP (RpoC-PAmCherry), and (C) ribosomal protein S1 (S1-PAmCherry) molecules relative to the nucleoid. Left to right: transmitted light image (scale bar, 1 μm), SytoGreen-stained nucleoid DNA with a segmented cell outline, and maps of mobile (blue) and immobile (red) molecule tracks. Histograms show localizations and SytoGreen fluorescence profiles projected onto the long cell axis (green line). (D) Average spatial distributions of Pol1, RNAP, and ribosomal protein S1 immobile and mobile molecules. See also Figure S1.

We examined the spatial distribution of mobile DNA-binding proteins relative to the nucleoid. As an example, we tracked Pol1-PAmCherry and RNAP-PAmCherry in live cells that were stained with SytoGreen dye to label DNA (Figures 2A and 2B). The positions of mobile molecules closely overlapped with the nucleoid in individual cells. Similarly, when averaged over many cells, the spatial distribution of the mobile population of molecules clearly demarcates the nucleoid shape (Figure 2D). This was in contrast to ribosomal protein S1, which has no direct DNA affinity. Consistent with previous reports (Sanamrad et al., 2014), the slow-moving S1 molecules, which are presumably incorporated into ribosomes, resided outside of the nucleoid area, whereas the mobile unincorporated subunits were distributed uniformly throughout the cell (Figures 2C and 2D). We hypothesized that the enrichment of mobile DNA-binding proteins in the nucleoid is caused by transient interactions with DNA during the target search process. The computation of D^∗^ values is based on the average movement of a molecule over a series of frames (here, 5 frames, 75 ms). The observed mobility may thus reflect a time average of the diffusion coefficient where 3D diffusion is interrupted by single or multiple transient DNA binding events with a duration of less than 75 ms. Consistent with this view, we observed that the mobility of DNA-binding proteins increased and tracks spread throughout the cell cytoplasm after treatment with the antibiotic rifampicin, which causes decompaction of the nucleoid (Figure S1D; Cabrera et al., 2009; Dworsky and Schaechter, 1973; Stracy et al., 2015). Nucleoid decompaction increases the space between DNA strands, which is expected to extend the intervals of 3D diffusion between transient DNA binding events.

### Generating chromosome-free cells to study protein diffusion in the absence of DNA

**Figure 3:**
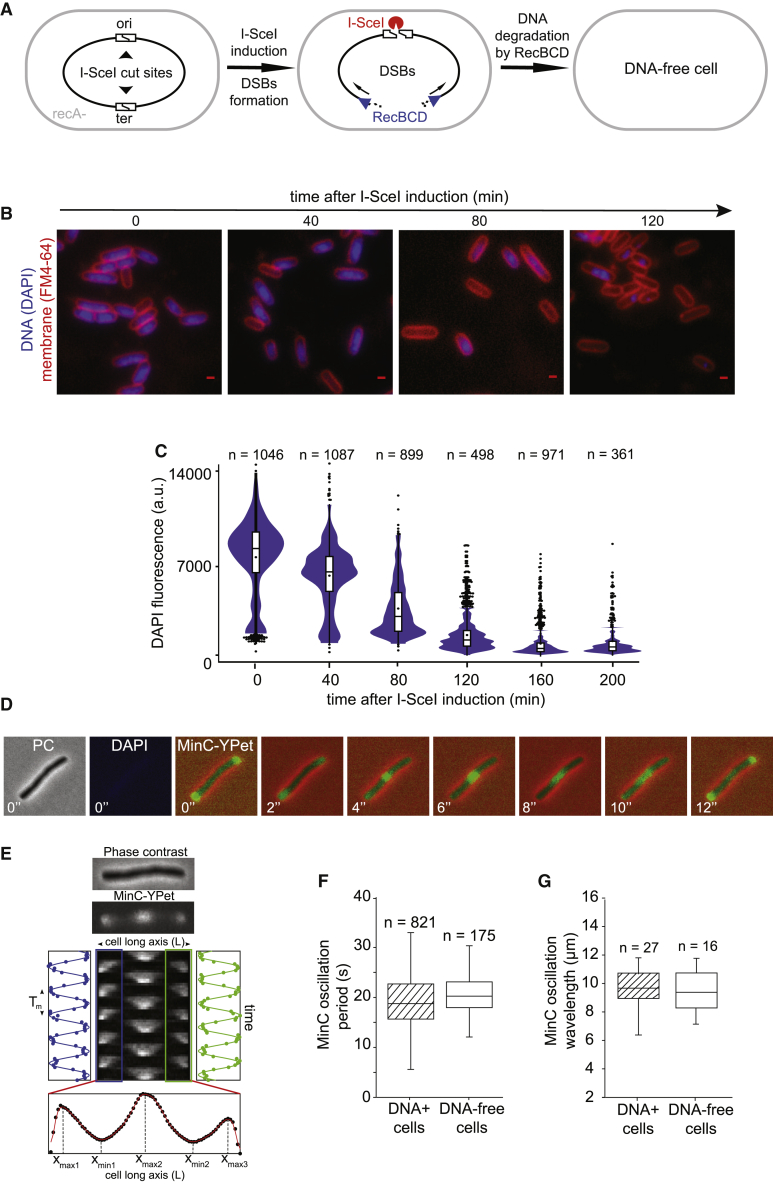
Generating chromosome-free cells that remain metabolically active (A) Schematic of the chromosome-degradation system. Induction of I-SceI endonuclease causes 2 double-stranded-breaks (DSBs) in *I-SceI* cut sites at diametrically opposed positions on the chromosome. In a *recA* strain, processing of DSBs by RecBCD results in complete chromosome degradation. (B) Chromosome degradation after I-SceI induction, revealed by loss of DAPI-stained DNA fluorescence (blue) in cells with an FM464-labeled membrane (red). Scale bar, 1 μm. (C) DAPI fluorescence profiles show complete chromosome degradation 120 min after I-SceI induction (black dot, mean and outliers; horizontal lines, median, first and third quartiles; n, number of cells analyzed). (D) MinC-YPet oscillation in an example chromosome-free cell. Cell filamentation was induced by cephalexin treatment. Transmitted light, DAPI, and MinC-YPet fluorescence images were obtained 120 min after I-SceI induction. Scale bar, 1 μm. (E) Kymograph of MinC-YPet oscillation in an example filamentous cell. Kymograph width corresponds to the long cell axis (L). Time-dependent intensity in the cell halves (blue, green) shows the oscillation period T_m_. The time-average profile underneath shows the oscillation wavelength. (F and G) MinC-YPet oscillation period and wavelength are similar with and without chromosome degradation (n, number of cells analyzed, error bars: standard deviation [STD]). See also Figures S2–S5.

The association of mobile DNA-binding proteins with the nucleoid could reflect genuine DNA-binding activity or be the result of a sieving effect, where protein movement is slowed in the nucleoid by physical entrapment in the mesh of DNA strands. Both of these interpretations are consistent with the increased mobility caused by nucleoid decompaction. To distinguish between the effects of sieving and non-specific DNA interactions, we compared the mobility of DNA-binding and non-DNA binding proteins in cells with and without DNA. We devised a method that removes all chromosomal DNA from cells while retaining the same cell size and intracellular protein concentration. We used the I-SceI endonuclease from *Saccharomyces cerevisiae* (Monteilhet et al., 1990), which introduces site-specific double-stranded breaks (DSBs) at *I-SceI* cut sites (*I-SceI*^*cs*^) inserted into the *E. coli* chromosome (Figure 3A; Lesterlin et al., 2014; Meddows et al., 2004). In the absence of RecA, which is essential for homologous recombination, creation of DSBs by I-SceI results in complete degradation of the chromosome by the RecBCD helicase-nuclease complex, a phenomenon referred to as *reckless* chromosome degradation (Skarstad and Boye, 1993; Willetts and Clark, 1969). To maximize the degradation rate, we inserted two cut sites on opposite sides of the chromosome, one close to the origin of replication and one in the *terminus* region (referred to as the OT strain) (Figure 3A). The *recA* gene was then inactivated in these strains (referred to as OT*recA-*), and degradation was triggered by expression of the plasmid-borne *I-SceI* gene under the control of an arabinose-inducible promoter. Chromosome degradation after *I-SceI* induction resulted in progressive disappearance of DAPI-stained DNA from cells (Figure 3B), which was complete within 120–160 min in most (∼92%) cells (Figures 3C, S2A, and S2B). This reflects the time required for 4 RecBCD complexes to degrade approximately one quarter of the chromosome (∼1,150 kb) from the 4 DNA ends generated by 2 DSBs at a speed of ∼160 bp/s, consistent with previous results (Lesterlin et al., 2014). A fraction of cells (∼8%) did not exhibit complete chromosome loss after 120 min (Figures S2A and S2B), likely because of heterogeneous induction of I-SceI from the arabinose-inducible promoter in the cell population (Siegele and Hu, 1997) or because of the limited number of RecBCD molecules per cell (Lepore et al., 2019). To ensure that our results reflected completely chromosome-free cells, we excluded cells that showed any remaining fluorescent DNA stain from our analysis.

### Chromosome-free cells remain metabolically active for several hours

Because protein mobility is influenced by the metabolic state of the cell (Parry et al., 2014), we explored whether cells remained metabolically active after chromosome degradation using two independent assays. First, to test whether ATP-driven mechanisms were affected by chromosome loss, we turned to the well-characterized Min system, which determines location by generating dynamic pole-to-pole oscillation of MinC, which is highly sensitive to ATP concentrations (Hu et al., 2002; Lutkenhaus, 2007). These oscillations are particularly striking in cells that have been grown into long filaments by treatment with the antibiotic cephalexin (Raskin and de Boer, 1999). We found that the oscillation period of fluorescent MinC-Ypet was ∼17 s with a wavelength of ∼10 μm in unperturbed cells and after chromosome degradation (Figures 3D–3G and S3), demonstrating that ATP concentration in chromosome-free cells remained stable for at least 2 h. Our results also indicate that the nucleoid has no influence on Min protein dynamics, in contrast to a previous report indicating that the oscillations may be coupled to chromosome segregation (Di Ventura et al., 2013).

Second, to test whether protein synthesis activity was maintained after chromosome loss, we used a non-degraded plasmid producing a reporter protein from a *P*_*lac*_ promoter. We found that IPTG-induced protein production continued for ∼200 min after I-SceI induction (Figure S4A). These tests establish that our chromosome degradation strategy is appropriate to study protein diffusion in metabolically active chromosome-free cells. We further confirmed that protein diffusion was not affected by inactivation of RecA per se or by induction of I-SceI in cells that do not contain any I-SceI cut sites, nor by DSB creation in RecA+ DNA repair-proficient cells (Figures S4B and S4C).

### The mobility of the Lac repressor increases in DNA-free cells

**Figure 4:**
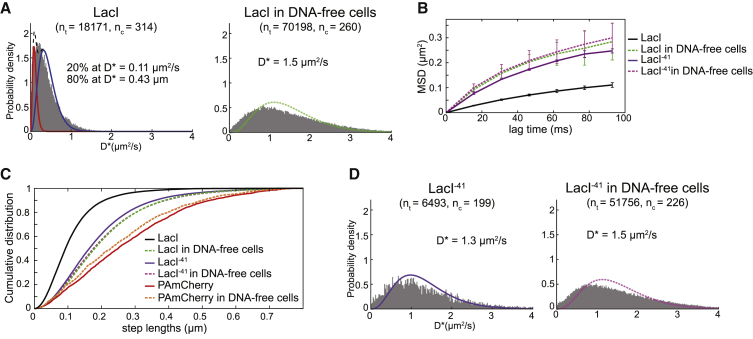
Diffusion of the lac repressor increases in chromosome-free cells (A) D^∗^ histograms of LacI-PAmCherry in unperturbed cells (left) fitted with a two-species model (black dashed line) of a mixture of immobile (red) and mobile molecules (blue) and D^∗^ distribution of LacI-PAmCherry in chromosome-free cells 120 min after I-SceI induction (right) fitted with a model for mobile molecules (green). (B) Mean-squared displacement plots from data in (A) and (D) ( Error bars: STD). (C) Cumulative distributions of the step lengths between consecutive localizations in unperturbed and chromosome-free cells for LacI-PAmCherry, the LacI^41^-PAmCherry DNA-binding mutant, and unconjugated PAmCherry. Distributions shift to longer steps with increasing diffusion coefficient. (D) D^∗^ histograms of LacI^41^-PAmCherry in unperturbed cells (left, purple) and in chromosome-free cells 120 min after I-SceI induction (right, magenta) fitted with a model for mobile molecules. See also Figure S5.

To test the effect of chromosome loss on intracellular diffusion, we first focused on the lac repressor (LacI) as a prototypical DNA-binding protein that searches for operator sequences by facilitated diffusion involving frequent non-specific DNA binding, sliding, and hopping (Elf et al., 2007; Garza de Leon et al., 2017; Hammar et al., 2012; Kao-Huang et al., 1977; Marklund et al., 2020). Chromosome degradation ∼120 min after I-SceI induction drastically changed the diffusion behavior of LacI-PAmCherry (Figure 4A). We no longer detected any immobile molecules; further, the mobility of the diffusing population increased significantly (from D^∗^ = 0.43 μm^2^/s in unperturbed cells to D^∗^ = 1.5 μm^2^/s in chromosome-free cells). The change in diffusion pattern is apparent in the D^∗^ distribution (Figure 4A), MSD curves (Figure 4B), and cumulative distributions of displacements per frame (Figure 4C). Cells with incomplete chromosome degradation were excluded based on their DNA staining signal (Figures S5A and S5B). The strong influence of the presence of the chromosome on LacI mobility could be due to DNA binding and sliding or a result of a general molecular sieving effect, where protein motion is hindered because of entrapment in the chromosome meshwork. The latter effect should influence the motion of all proteins in the cell, even those that have no DNA affinity. To test this directly, we imaged a truncated LacI^−41^ mutant with most of its DNA-binding domain (41 N-terminal amino acids) removed. For this mutant, all specific and non-specific DNA binding modes are abolished (Elf et al., 2007; Garza de Leon et al., 2017); hence, it shows essentially no immobile molecules (Figure 4D). Notably, LacI^−41^ also had a much higher D^∗^ than the mobile population of wild-type LacI (D_LacI−41_^∗^ = 1.3 μm^2^/s versus D_LacI_^∗^ = 0.43 μm^2^/s). This difference far exceeded the 2%–3% change expected solely from the 9-kDa decrease in protein size because of the truncation (considering D ∼M^−1/3^). After chromosome degradation, LacI^−41^ only showed a small increase in mobility (from D^∗^ = 1.3 μm^2^/s to 1.5 μm^2^/s) (Figures 4B–D). To test whether this is general, we also measured the diffusion of unconjugated PAmCherry alone and found no significant change between unperturbed and chromosome-free cells (Figure 4C). Therefore, the presence of the chromosome has only a minor influence on diffusion of a protein that has no affinity for DNA. This is consistent with observation that other fluorescent proteins diffuse in the whole cell volume with no evidence that the nucleoid slows their motion (Bakshi et al., 2011; English et al., 2011). These data do not exclude the possibility that DNA sieving may hinder the movement of proteins and macromolecular complexes that are much larger than LacI and fluorescent proteins, such as 70S ribosomes, which are occluded from the nucleoid (Figure 2C; Sanamrad et al., 2014).

### Transient DNA interactions strongly affect the mobility of diverse DNA-binding proteins

**Figure 5:**
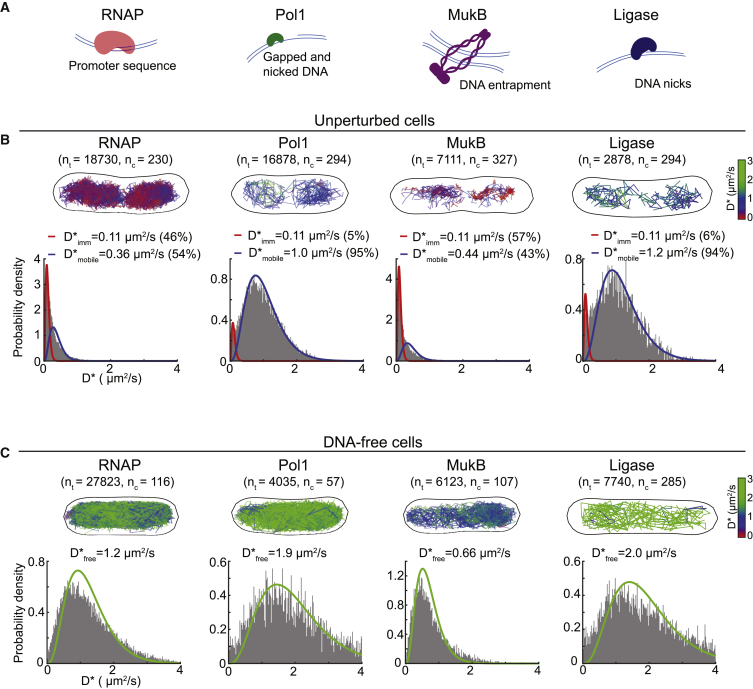
Chromosome degradation increases the mobility of diverse types of DNA-binding proteins (A) DNA-binding modes of RNAP, Pol1, MukB, and ligase. (B) Tracks of RNAP-PAmCherry, Pol1-PAmCherry, MukB-PAmCherry, and LigA-PAmCherry in example cells, with the color of each track representing its D^∗^ value. Also shown are D^∗^ histograms in unperturbed cells, fitted with a two-species model of a mixture of immobile (red) and mobile (blue) molecules. (C) Tracks of RNAP-PAmCherry, Pol1-PAmCherry, MukB-PAmCherry, and LigA-PAmCherry in example chromosome-free cells 120 min after I-SceI induction, with the color of each track representing its D^∗^ value. D^∗^ histograms in chromosome-free cells were fitted with a single-species model for mobile molecules.

Having established that chromosome degradation increases the mobility of LacI primarily because of loss of DNA interactions, we wanted to find out whether this was generally the case for diverse types of DNA-binding proteins. We chose four proteins representing distinct types of DNA interactions (Figure 5A): RNAP recognizes specific promoter sequences and transcribes genes (Mazumder and Kapanidis, 2019); DNA polymerase I (Pol1) performs DNA synthesis at gapped or nicked DNA sites (Joyce and Steitz, 1994); the SMC protein MukB interacts non-specifically with DNA to aid chromosome segregation (Nolivos et al., 2016; Reyes-Lamothe et al., 2012; Rybenkov et al., 2014); and ligase (LigA) interacts with DNA nicks and catalyzes joining of DNA ends (Shuman, 2009). These proteins not only have different biological functions but cover a broad range of shapes, molecular weights, oligomeric states, and intracellular concentrations (Table 1).

**Table 1:** Quantitative partitioning of DNA-binding protein activity

Protein	Function	Size of PAmCherry-Labeled Protein or Protein Complex (kDa)^a^	Molecules/Cell	D^∗^_mobile_ (μm^2^/s)	D_free_ (μm^2^/s)	% Long-Lived DNA-Bound Molecules	% Transient DNA- Bound Molecules	% Freely Diffusing Molecules	Copy Number Reference
MukBEF (MukB subunit)	chromosome organization	1,006 (dimer of dimers)	100	0.44 (0.43–0.46)	1.2 (1.1–1.3)	58 (56–61)	24 (21–25)	18 (17–20)	Badrinarayanan et al., 2012
RNA polymerase (β’ subunit)	transcription	478 (holoenzyme)	4,000	0.36 (0.36–0.37)	2.7 (2.5–2.8)	45 (43–46)	48 (46–50)	7 (68)	Bakshi et al., 2013; Stracy et al., 2015
DNA gyrase (GyrA subunit)	supercoiling	424 (heterotetramer)	600	0.35 (0.34–0.36)	2.4^b^ (0.7–4.2)	55 (53–57)	38^b^ (29–47)	7^b^ (0–15)	Stracy et al., 2019
Topoisomerase IV (ParC subunit)	supercoiling/decatenation	366 (heterotetramer)	80	0.40 (0.39–0.41)	2.7^b^ (1.0–4.5)	37 (35–40)	52^b^ (41–65)	11^b^ (0–20)	Zawadzki et al., 2015
UvrA	DNA repair	270 (dimer)	80	0.36 (0.33–0.38)	3.4^b^ (1.7–5.1)	43 (42–46)	51^b^ (43–58)	6^b^ (0–12)	Stracy et al., 2016
MutS	DNA repair	248 (dimer)	100	0.37 (0.35–0.39)	3.7^b^ (1.9–5.5)	26 (24–29)	68^b^ (63–76)	6^b^ (0–9)	Uphoff et al., 2016
Lac repressor (LacI)	gene regulation	220 (tetramer)	40	0.40 (0.39–0.43)	3.3 (3.1–3.5)	41 (37–46)	55 (50–60)	4 (3–5)	Garza de Leon et al., 2017
DNA polymerase 1 (PolA)	DNA repair/replication	128 (monomer)	500	1.04 (1.02–1.07)	6.6 (6.5–6.7)	4 (3–4)	58 (56–64)	37 (33–41)	Uphoff et al., 2013
DNA ligase (LigA)	DNA repair/replication	102 (monomer)	100	1.18 (1.11–1.23)	6.9 (6.5–7.1)	7 (4–9)	56 (52–59)	37 (35–41)	Uphoff et al., 2013
Histone-like nucleoid structuring protein (H-NS)	nucleoid associated protein/gene regulation	87 (dimer)	20,000	0.41 (0.40–0.42)	8.0^b^ (5.8–10.0)	73 (71–75)	27^b^ (25–29)	0.1^b^ (0–1)	Katayama et al., 1996
Heat-unstable protein (HU)	nucleoid associated protein/gene regulation	48 (heterodimer)	30,000	0.33 (0.32–0.34)	12.6^b^ (7.9–17.4)	23 (21–24)	77^b^ (76–79)	0.4^b^ (0–1)	Gruber, 2014

**Figure 6:**
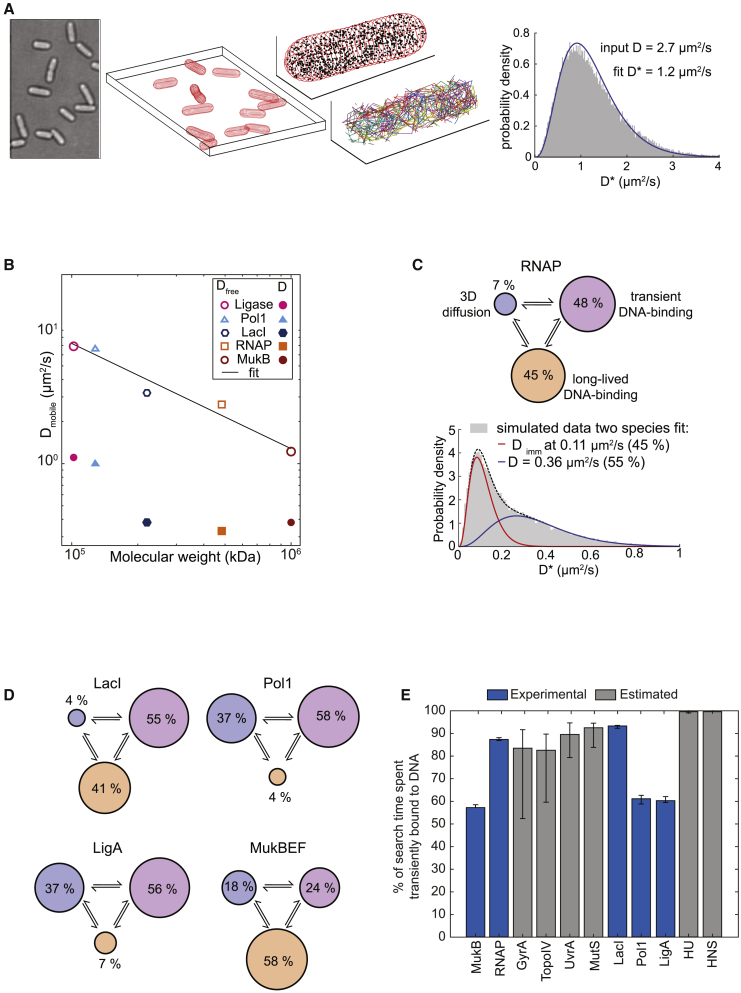
Quantitative partitioning of protein states (A) Illustration of Brownian motion simulation to estimate the unbiased diffusion coefficients D_mobile_ (in unperturbed cells) and D_free_ (in chromosome-free cells). (B) D_mobile_ and D_free_ plotted versus molecular weight M on a log scale. Linear fit log(D_free_) = α∙log(c∙M). (C and D) Partitioning long-lived DNA-binding (orange), transient DNA-binding (purple), and 3D diffusion (blue) states for RNAP and (D) for LacI, Pol1, LigA, and MukB. (E) The percentage of search time spent bound non-specifically to DNA for all 11 studied DNA-binding proteins. Blue bars show the proteins with D_free_ measured in chromosome-free cells, and gray bars show proteins with D_free_ estimated from the fit in (B). Error bars: STD. See also Figure S6.

Brackets indicate 95% confidence bounds.

a In some cases, the functional complex contains more than one copy of the subunit that was labeled, and the complex therefore contains more than one PAmCherry protein.

b Predicted values based on extrapolation of D_free_ values shown in Figure 6.

Considering the differences in the function and physical characteristics of RNAP, Pol1, MukB, and LigA, any shared aspects of their diffusion behavior likely indicate universal mechanisms of the DNA target search. In unperturbed cells, a large fraction of RNAP-PAmCherry and MukB-PAmCherry molecules were immobile or diffusing slowly (RNAP, D^∗^ = 0.36 μm^2^/s; MukB, D^∗^ = 0.39 μm^2^/s), whereas Pol1-PAmCherry and LigA-PAmCherry molecules were rarely immobile for the entire trajectory and diffused faster (D^∗^ = 1.0 μm^2^/s and D^∗^ = 1.2 μm^2^/s, respectively) (Figure 5B), consistent with our previous observations (Badrinarayanan et al., 2012; Stracy et al., 2015; Uphoff et al., 2013). Despite the differences in the diffusion profiles, a unifying feature was the clear nucleoid association for all three proteins in unperturbed cells (Figures 2 and 5B). Chromosome degradation had the same effect for all four proteins (compare Figures 5B and 5C): the populations of long-lived immobile molecules disappeared, and diffusion of the mobile proteins increased substantially (RNAP, D^∗^ = 1.2 μm^2^/s; Pol1, D^∗^ = 1.9 μm^2^/s; MukB, D^∗^ = 0.66 μm^2^/s; LigA, D^∗^ = 2 μm^2^/s) (Figure 5C). Furthermore, the tracks filled the entire cytoplasm of chromosome-free cells (Figure 5C). These results match our observations for the Lac repressor (Figure 4), and taken together, they demonstrate that transient DNA interactions dictate the mobility and spatial distribution of diverse types of DNA-binding proteins.

### The mobility of DNA-binding proteins shows a steep size dependence in chromosome-free cells

Accurate quantification of diffusion coefficients from single-molecule tracking experiments requires consideration of several biases, such as localization error and confinement in the cell volume (English et al., 2011; Uphoff, 2016). To determine unbiased D values from experimentally measured D^∗^, we applied stochastic Brownian motion simulations to generate artificial single-molecule tracks using an identical number of molecules in the same segmented 3D cell volumes as in the experimental data (Figure 6A). Localization error and stochastic disappearance of tracks because of photobleaching were also modeled, resulting in the same sampling and biases as in the experiments. We determined an unbiased estimate of the diffusion coefficient D from the best match (according to a least-squares metric) between measured D^∗^ distributions and those obtained from simulations with a range of input diffusion coefficients.

Using this procedure, we estimated the mean unbiased diffusion coefficients of LacI, RNAP, Pol1, LigA, and MukB molecules after chromosome degradation (D_free_ values in Table 1). We also performed single-molecule tracking at 3-fold shorter camera exposure times (5 ms) and verified that the corresponding simulation results were robust with regard to the data acquisition and simulation parameters (Figure S6). In contrast to the results obtained in unperturbed cells (D_mobile_), we found a clear inverse relationship between the mass and the diffusion coefficient of DNA-binding in chromosome-free cells (D_free_) (Figure 6B). Fitting a power-law D_free_ = c∙M^α^ yielded an exponent of α = −0.75, showing that protein mobility decreases more steeply with increasing mass than predicted by the Stokes-Einstein model (α = −0.33). Therefore, the crowded cytoplasm has sieving properties even in the absence of the chromosome meshwork. Indeed, diffusion of DNA-binding proteins in DNA-free cells shows a similar mass dependence as cytoplasmic proteins that have no DNA-binding function in unperturbed cells (α = −0.7) (Mika and Poolman, 2011).

### Transient DNA-binding events dominate the target search

Comparison of the diffusion coefficient in unperturbed cells versus chromosome-free cells allows quantification of the contribution of non-specific DNA interactions to the observed mobility of DNA-binding proteins during the target search. By simulating molecules rapidly interconverting between freely diffusing (with D_free_ from chromosome-free cells) and DNA-bound (D_bound_ = 0.04 μm^2^/s), we can establish the fraction of time a protein spends transiently bound to DNA, Φ_transient_binding_, which best recapitulates the observed mobility of mobile molecules during their target search, as measured in unperturbed cells (D^∗^_mobile_; Figure 1B). Using this approach, we find that Φ_transient_binding_ is greater than 0.5 for RNAP, Pol1, MukB, LigA and LacI, demonstrating that they spend the majority of their search process bound non-specifically to DNA (Φ_transient_binding_ RNAP, 87%; Pol1, 61%; MukB, 58%; LigA, 60%; LacI, 93%) (Figure 6C). The value for LacI is in good agreement with a previous estimate (Elf et al., 2007). Including the populations of long-lived immobile molecules measured in unperturbed cells (representing molecules likely to be bound at specific DNA target sites; Figure 1) in the calculations, the total percentage of DNA-bound molecules at any time is even higher (RNAP, 93% [95% confidence interval [CI] = 89–96]; LacI, 96% [87–100]; Pol1, 63% [59–68]; MukB, 82% [77–86]; LigA, 63% [57–68]) (Figure 6D). Based on these results, we report quantitative partitioning of DNA-binding proteins into three distinct states of mobility: long-lived specific binding at DNA target sites, transient non-specific DNA-binding, and free diffusion between DNA strands (Figures 6C and 6D).

Using the estimate of α = −0.75 to extrapolate D_free_ and the measured percentage of long-lived DNA binding and the measured D^∗^_mobile_ values, we performed the same partitioning of diffusive states for all DNA-binding proteins considered in this study (Table 1). In all cases, the fraction of the target search spent bound non-specifically to DNA was more than 50%, and for the small nucleoid-associated protein HU, this estimated fraction was as high as 99%.

## Discussion

Our study demonstrates the ubiquity of transient non-specific DNA interactions for diverse DNA-binding proteins *in vivo*. Despite their different sizes, DNA targets, mobility, and copy numbers in the cell, the target search of all DNA-binding proteins examined here is dominated by transient non-specific DNA binding. Considering such widespread and frequent non-specific DNA interactions of all types of DNA-binding proteins, an important question is how these, in turn, affect DNA transactions.

Our analysis shows that the chromosome DNA mesh is not a physical barrier for the intracellular motion of proteins (at least up to a molecular weight of 100 kDa). In fact, mobile DNA-binding proteins (even large complexes such as RNAP) are enriched in the densest regions of the nucleoid by frequent non-specific DNA interactions. These results demonstrate that the apparent mobility of DNA-binding proteins depends on DNA-binding activity rather than molecular weight, as concluded before (Kumar et al., 2010). Although we have found no evidence of a nucleoid sieving effect for DNA-binding proteins during their target search, previous reports have established that large macromolecular complexes that do not bind DNA, such as protein aggregates, 70S ribosomes, and MS2-RNA systems, are excluded from the nucleoid (Landgraf et al., 2012; Lindner et al., 2008; Sanamrad et al., 2014; Stracy et al., 2015; Stylianidou et al., 2014). These findings are consistent with the view that DNA-interacting proteins can diffuse freely in the whole cell compartment and are enriched in the nucleoid volume because of frequent non-specific interactions with the DNA. Because of the limits of spatial and temporal resolution, we cannot resolve the precise mode of these DNA interactions, but it is likely that, for some proteins, these transient binding events are predominantly 1D sliding, whereas for others, they are hopping or intersegmental transfer. Although the target search is dominated in time by non-specific binding, the explored space is determined by the intervals of 3D diffusion between the binding events.

For example, LacI slides an average distance of only 45 bp during a binding event, and its 1D diffusion coefficient on DNA is 2 orders of magnitude smaller than its D_free_ (Elf et al., 2007; Hammar et al., 2012). Protein hopping or sliding along the DNA can enhance the search efficiency for any individual protein, whereas overcrowding the chromosome with non-specifically bound proteins would globally reduce the search kinetics because of obstruction of target sites and sliding collisions (Li et al., 2009). This trade-off likely influenced the evolution of protein abundances and their non-specific DNA binding affinities. According to theory, the rate of target encounter is maximized when a searching protein spends equal time in solution and non-specifically bound to DNA (von Hippel and Berg, 1989). Experiments on LacI have offered an explanation why, in reality, the target search is dominated by non-specific binding (Hammar et al., 2012). According to that study, LacI often slides past its target site without engaging in stable binding. When the probability of successful binding upon target encounter is low, the optimal search strategy requires more non-specific binding and less time in solution (Hammar et al., 2012). Notably, the close association of proteins with the nucleoid may constrain their motion in ways that reduce the dimensionality of their target search in 3D space. This can affect the search kinetics so that the starting position of a protein in the cell affects the average time it will take to reach its target (Bénichou et al., 2010). In contrast to a random 3D search in dilute solution, a protein performing a so-called “compact” search process densely samples its crowded environment and is more likely to revisit the same target multiple times than reach other distant sites. This has been demonstrated for a transcription factor in the eukaryotic nucleus (Izeddin et al., 2014) and may lead to effects like transcriptional bursting (Meyer et al., 2012).

Given the diversity of the proteins we tested, their target-specific DNA interactions are likely to be very different from each other, suggesting that a more universal interaction plays the largest role in the frequent non-specific attraction during the target search. We speculate that the electrostatic interaction between positively charged functional groups on the surface of the proteins and the largely invariant negatively charged phosphate backbone of the DNA may drive this phenomenon (Kalodimos et al., 2004; Redding and Greene, 2013). Indeed, the surface charge of proteins strongly affects their mobility in cells (Elowitz et al., 1999; Schavemaker et al., 2017), and high intracellular salt concentrations can disrupt DNA binding *in vivo* (Cagliero and Jin, 2013). Although positively charged proteins have been shown to transiently bind ribosomes (Schavemaker et al., 2017), multiple aspects of our data show that DNA binding is by far the dominant interaction mode. For example, DNA binding proteins are rarely located in the ribosome-rich cell endcaps, and degrading the DNA significantly increases the mobility of DNA-binding proteins despite ribosome subunits still being present.

The abundance of non-specific binding also suggests that the percentage of the chromosome occupied by proteins is high. Based on the combined percentage of DNA-bound proteins (specific and non-specific) shown in Table 1 and literature estimates of their copy number and DNA footprint, we estimate that, at any given time, 28% of the chromosome is occupied by the 11 studied proteins (12% long-lived binding and 16% transient binding; STAR methods). These proteins represent just a fraction of all DNA-binding proteins, suggesting that the total DNA occupancy of the entire proteome is substantially higher, highlighting the importance of studying protein-DNA interactions in the native cellular environment. Besides the target search, non-specific DNA binding likely also influences the dissociation of proteins from their specific target sites. Several studies have shown that competition with proteins in solution accelerates DNA unbinding because of invasion of a partially dissociated state (Chen et al., 2015; Gibb et al., 2014; Graham et al., 2011; Loparo et al., 2011). Although this has been demonstrated for exchanges between identical proteins in solution and on DNA, the overwhelming abundance of other DNA-binding proteins and their frequent transient associations with DNA likely contribute significantly to the turnover of DNA-bound proteins *in vivo*. Thus, non-specific DNA interactions play a crucial role in the search and dissociation of DNA-binding proteins. Growing evidence suggests that protein condensation via liquid-liquid phase separation (LLPS) enables rapid and reversible compartmentalization of protein activity in cells. Although the phenomenon appears to be less prevalent in bacteria than in eukaryotes, several bacterial nucleic acid-binding proteins, including RNAP, have been shown to form condensates indicative of LLPS (Al-Husini et al., 2018; Harami et al., 2020; Hondele et al., 2019; Ladouceur et al., 2020). To what extent such mechanisms may contribute to DNA target search processes remains unknown. The chromosome degradation method presented here could help to resolve functional interdependencies between the nucleoid and formation of protein condensates.

Beyond these fundamental implications, our system for generating chromosome-free cells has broader potential applications in synthetic biology. Alternative approaches, such as minicells, which are generated by forcing aberrant cell divisions close to the cell poles, have a perturbed makeup of proteins and contain few DNA-binding proteins (Shepherd et al., 2001). In contrast, our chromosome-degraded cells retain the DNA binding proteins and keep the same cell size and geometry. Moreover, the chromosome-degraded cells maintain ATP levels and can produce plasmid-encoded proteins for several hours, enabling targeted expression of exogenous genes without interference from chromosomal gene expression. Removing all endogenous gene circuitry from *E. coli* cells but maintaining the transcription machinery provides customizable non-viable containers for a range of applications, including expression of synthetic gene circuits, biosensing, and drug delivery (Caliando and Voigt, 2015; Fan et al., 2020; MacDiarmid et al., 2007; Rampley et al., 2017).

### Limitations

The limited temporal resolution of single-molecule tracking experiments prevents direct observation of transient non-specific DNA binding events. To overcome this issue, we devised an alternative approach to estimate the relative fraction of time spent bound to DNA and diffusing. Although we cannot determine exact DNA binding times, our data show that proteins typically interconvert multiple times between bound and diffusing states during the 75-ms duration per track. For some proteins, such as HU, the difference between specific and non-specific binding is indistinct. Last, we draw general conclusions from our measurements of a set of 11 diverse DNA-binding proteins. However, we cannot exclude the possibility that other types of DNA-binding proteins behave differently from those we studied.

## STAR★Methods

### Key resources table

**Table:** 

REAGENT or RESOURCE	SOURCE	IDENTIFIER
**Bacterial strains**		

MG1655 F- lambda- *ilvG*- *rfb*-50 *rph*-1	Coli Genetic Stock Centre	CGSC#: 7740
*TB28 I-SceI*^*CS*^*-ilvA*	Bernhardt and de Boer, 2005	N/A
TB28 *I-SceI*^*CS*^*-ilvA-FRT* (3953 kb)	This study	N/A
TB28 *I-SceI*^*CS*^*-ydeO*	This study	N/A
TB28 *I-SceI*^*CS*^*-ydeO-FRT-cat-FRT* (1580 kb)	This study	N/A
TB28 *I-SceI*^*CS*^*-ilvA-FRT, I-SceI*^*CS*^*-ydeO-FRT* (this strain is referred to as OT hereafter)	This study	N/A
RNAP-PAmCherry (MG1655 *rpoC-PAmCherry-FRT-kan-FRT)*	Stracy et al., 2015	N/A
HU-PAmCherry (MG1655 *hupB-PAmCherry-FRT-kan-FRT)*	Stracy et al., 2015	N/A
HN-S-PAmCherry (MG1655 *Hns-PAmCherry-FRT-kan-FRT)*	Stracy et al., 2015	N/A
FIS-PAmCherry (MG1655 *fis-PAmCherry-FRT-kan-FRT)*	Uphoff et al., 2013	N/A
LacI-PAmCherry (MG1655 *LacI-PAmCherry)*	Garza de Leon et al., 2017	N/A
Pol1-PAmCherry (MG1655 *polA-PAmCherry-FRT-kan-FRT)*	Uphoff et al., 2013	N/A
LigA-PAmCherry (MG1655 *ligA-PAmCherry-FRT-kan-FRT)*	Uphoff et al., 2013	N/A
UvrA-PAmCherry MG1655 *uvrA-PAmCherry-FRT-kan-FRT*	Stracy et al., 2016	N/A
MutS-PAmCherry (MG1655 *mutS-PAmCherry-FRT-kan-FRT)*	Uphoff et al., 2016	N/A
TopoIV-PAmCherry (MG1655 *parC-PAmCherry-FRT-kan-FRT)*	Zawadzki et al., 2015	N/A
MukB-PAmCherry (MG1655 *mukB-PAmCherry-FRT-kan-FRT)*	Badrinarayanan et al., 2012	N/A
GyrA-PAmCherry (MG1655 *gyrA-PAmCherry-FRT-kan-FRT)*	Stracy et al., 2019	N/A
*recA- strain (*TB28 *recAT233C-Tet)*	Lesterlin et al., 2014	N/A
MinC-Ypet (*minC-Ypet)*	Bisicchia et al., 2013	N/A
OT RNAP-PAmCherry (OT *rpoC-PAmCherry-FRT-kan-FRT)*	This study	N/A
OT Pol1-PAmCherry (OT *polA-PAmCherry-FRT-kan-FRT)*	This study	N/A
OT LigA-PAmCherry (OT *ligA-PAmCherry-FRT-kan-FRT)*	This study	N/A
OT MukB-PAmCherry (OT *mukB-PAmCherry-FRT-kan-FRT)*	This study	N/A
OT LacI-PAmCherry (OT */ p lacI-PAmCherry)*	This study	N/A
OT LacI^41^-PAmCherry (OT */ p lacI*^*41*^*-PAmCherry)*	This study	N/A
OT Free PAmCherry (OT pBAD\HisB PAmCherry1)	This study	N/A
OT FIS-PAmCherry (OT *fis-PAmCherry-FRT-kan-FRT)*	This study	N/A
OT *recA- (*OT *recAT233C-Tet)*	This study	N/A
OT RNAP PAmCherry *recA- (*OT *rpoC-PAmCherry-FRT-kan-FRT recAT233C-Tet)*	This study	N/A
OT Pol1-PAmCherry *recA- (*OT *polA-PAmCherry-FRT-kan-FRT recAT233C-Tet)*	This study	N/A
OT LigA-PAmCherry *recA- (*OT *ligA-PAmCherry-FRT-kan-FRT recAT233C-Tet)*	This study	N/A
OT MukB-PAmCherry *recA- (*OT *mukB-PAmCherry-FRT-kan-FRT recAT233C-Tet)*	This study	N/A
OT LacI-PAmCherry *recA-* (OT */ P lacI-PAmCherry recAT233C-Tet)*	This study	N/A
OT LacI^41^-PAmCherry *recA- 9*OT */ P lacI*^*41*^*-PAmCherry recAT233C-Tet)*	This study	N/A
OT Free PA-mCherry *recA- (*OT pBAD\HisB PAmCherry1 *recAT233C-Tet)*	This study	N/A

**Chemicals, peptides, and recombinant proteins**		

EZ Rich Defined Medium RDM (Teknova Inc)	VWR	200059-658

**Deposited data**		

Raw and analyzed data	This study	http://data.mendeley.com/login?redirectPath=/datasets/4skb3txv92/draft?a=11f7a88d-70c3-43e0-9883-b1d313bc6c92

**Software and algorithms**		

MATLAB	MathWorks	https://uk.mathworks.com/products/MATLAB.html
NIS-Elements AR	Nikon	https://www.microscope.healthcare.nikon.com
ImageJ	Schneider et al., 2012	https://imagej.nih.gov/ij/
MicrobJ	Ducret et al., 2016	https://www.microbej.com
MicrobeTracker	Sliusarenko et al., 2011	http://microbetracker.org/

### Resource availability

#### Lead contact

Further information and requests for resources should be directed to the Lead Contact, Christian Lesterlin (Christian.lesterlin@ibcp.fr).

#### Materials availability

The strains generated in this study are available without restriction.

#### Data and code availability

Original PALM localization and tracking data are available at Mendeley data: http://dx.doi.org/10.17632/4skb3txv92.1. All materials and codes are available upon request.

### Experimental model and subject details

#### Bacterial strains, plasmids and growth

Bacterial strains and plasmids are listed in Table S2. All experiments were performed in *E. coli* TB28 background strain (MG1655 *ΔlacIZYA*) (Bernhardt and de Boer, 2005). PAmCherry fusion proteins expressed from their endogenous chromosome loci were previously characterized: RNAP, HU and HN-S (Stracy et al., 2015), LacI (Garza de Leon et al., 2017), Pol1 and LigA (Uphoff et al., 2013), UvrA (Stracy et al., 2016), MutS (Uphoff et al., 2016), ParC (Zawadzki et al., 2015), MukB (Badrinarayanan et al., 2012), GyrA (Stracy et al., 2019). Fusions were moved to *E. coli* TB28 strain by P1 transduction. Construction of plasmids expressing LacI-PAmCherry or LacI mutant are described in (Garza de Leon et al., 2017). Unconjugated PAmCherry was produced from the plasmid pBAD\HisB PAmCherry1 (Endesfelder et al., 2013). ParB-mCherry was produced from pSN70 plasmid (Nolivos et al., 2019). The *I-SceI* cut site (*I-SceI*^*CS*^) is followed by *cat* gene (chloramphenicol resistance) flanked by *frt* sites as described previously (Lesterlin et al., 2014). *I-SceI*^*CS*^ was inserted in two chromosome loci by λ-Red recombination (Datsenko and Wanner, 2000); *ilvA* (3953 kb) close to the origin of replication, and *ydeO* (1580 kb) in the *terminus* region. Using sequential P1 transduction, we constructed the OT strain (for *Ori-Ter*) carrying *ilvA::I-SceI*^*CS*^ and *ydeO::I-SceI*^*CS*^. After each transduction round, the *cat* gene was removed using Pcp20 plasmid (Datsenko and Wanner, 2000). P1 transduction was also used to transfer *recA-* mutation *recAT233C-Tet* or *minC-Ypet* allele (Bisicchia et al., 2013) alleles. Unless otherwise stated, cells were grown at 30°C in M9 medium supplemented with glucose (0.2%). When appropriate, growth media were supplemented with Ampicillin (Ap) 100 μg/ml, Chloramphenicol (Cm) 20 μg/ml or Kanamycin (Kn) 50 μg/ml.

### Method details

#### Sample preparation for microscopy

OT strains carrying two *I-SceI*^*CS*^ were transformed with pSN1 plasmid carrying the *I-SceI* gene under the control of the *P*_*lac*_ promoter and plated on LB agarose plates containing 0.2% glucose and ampicillin at 30°C. Transformant clones were propagated on LB agarose plates containing 0.2% glucose and ampicillin. Transformation was performed *de novo* before each experiment since strains carrying *I-SceI*^*CS*^ and the pSN1 plasmid exhibit genetic instability due to leaky *I-SceI* expression causing unrepairable DNA double-stranded breaks in the *recA-* strain. For each strain, a single colony was inoculated in M9 minimal medium supplemented with 0.2% glucose and ampicillin and incubated overnight at 30°C with agitation (140 rpm). The next day, overnight cultures were diluted and grown to early exponential phase (OD_600nm_ ∼0.2). 0.2% arabinose was added to induce the production of I-SceI endonuclease and initiate chromosome degradation in the *recA-* strains. Cultures were incubated at 30°C with agitation for the duration indicated in the text and Figures (120 min for complete DNA degradation) before microscopy. For control experiments in fixed cells, 2.5% paraformaldehyde was added to the growth media for 1 hour prior to imaging. Cell filamentation was induced by addition of cephalexin at final concentration of 5 μg/ml.

The cell suspension was concentrated by centrifugation (benchtop centrifuge at 6000 rpm), removal of the supernatant and resuspension in 1/10^th^ of the initial sample volume. Cells were immobilized on pads of 1% low-fluorescence agarose (Biorad) in M9 medium with 0.2% glucose as previously described (Lesterlin and Duabrry, 2016). For PALM microscopy 0.17 mm thickness coverslips were heated in an oven to 500°C to remove any background fluorescent particles before use. For quantification of chromosome degradation and MinC-Ypet oscillation by wide-field epifluorescence imaging, DNA staining was performed by incubating the cell suspension for 15 min with 2 4’,6-diamidino-2-phenylindole (DAPI) at 4 μg/ml prior to cell concentration and imaging. For multi-color imaging of the nucleoid and PAmCherry fusions, we stained DNA with 500 nM SytoGreen for 15 min before imaging (because DAPI excitation would cause photoactivation of PAmCherry).

#### Wide-field epifluorescence microscopy imaging

Wide-field epifluorescence microscopy imaging of DAPI-stained cells was carried out on an Eclipse Ti-E microscope (Nikon), equipped with 100 s/1.45 oil Plan Apo Lambda phase objective, Flash4 V2 CMOS camera (Hamamatsu), and using NIS Elements software for image acquisition. Acquisition was performed in phase contrast and epifluorescence mode using 50% power of a Fluo LED Spectra X light source at 405 nm and 560 nm excitation wavelengths for DAPI and ParB-mCherry, respectively.

Wide-field imaging of MinC-Ypet was carried out on a Nikon Eclipse TE2000-U microscope equipped with a 100X objective, CCD camera (Cool-SNAP by Photometrics) and Metamorph 6.2 acquisition software. Time-lapse movies were acquired in phase contrast and epifluorescence at 2 s intervals with 50 ms exposure for MinC-Ypet at 30°C.

#### Widefield epifluorescence image analysis

Cells were automatically detected using the MicrobeJ plugin for Fiji (Ducret et al., 2016; Schneider et al., 2012). Intracellular DAPI or ParB-mCherry mean fluorescence intensity (a.u.) was automatically extracted and plotted using the MicrobeJ results interface. For analysis of MinC oscillation, cells were outlined using the MATLAB-based tool MicrobeTracker (Sliusarenko et al., 2011). The fluorescence signal was integrated across the cross-section of each cell to generate a one-dimensional fluorescence profile in each frame. The fluorescence signal was normalized to the total fluorescence in each frame to remove photobleaching effects and facilitate MinC-Ypet localization analysis. The fluorescence signals obtained from each cell were further analyzed by generating kymographs using custom MATLAB code. The width of the kymograph corresponds to the cell length L. We integrated the fluorescence intensity for both cell halves at in each frame,

$$F_{x,left}\left(t\right)=\int_{x=0}^{L/2}f\left(x,t\right)dx\;F_{x,right}\left(t\right)=\int_{x=L/2}^{L}f\left(x,t\right)dx$$

By fitting the data to a trigonometric function, the oscillation period is calculated from the angular frequency ω=(2π/T)

$$F_{x,\frac{left}{right}}\left(t\right)=a\cdot \cos\left(\omega \cdot t\right)+b\cdot \sin\left(\omega \cdot t\right)$$

The time-averaged concentration profile of MinC is obtained by integration of the entire kymograph over all frames,

$$F_{t}\left(x\right)=\int_{t}f\left(x,t\right)dt$$

For analyzing MinC oscillations in filamentous cells, a slightly modified kymograph analysis was used. The MinC concentration profile was determined as described above. The positions of the fluorescence minima *xmin* were used to split the kymographs into several stripes. The overall oscillation period *Tm* was calculated as the average of all oscillation periods determined for each stripe in the kymograph. The oscillation wavelength was determined from the distance between two neighboring peaks. Depending on the length of the cell and the number of oscillations a set of wavelengths was determined from which a mean wavelength was calculated as

$$\lambda =\frac{1}{n}\sum_{i=1}^{n}\left(x_{min,i+1}-x_{min,i}\right)$$

Where the number *n* of oscillations corresponds to the number of peaks – 1.

#### Live-cell photoactivated single-molecule tracking

Live cell photoactivated single-molecule tracking was performed on a custom-built total internal reflection fluorescence (TIRF) microscope built around the Rapid Automated Modular Microscope (RAMM) System (ASI Imaging) as previously described (Uphoff, 2016). PAmCherry activation was controlled by a 405 nm laser and excited with 561 nm. All lasers were provided by a multi-laser engine (iChrome MLE, Toptica). At the fiber output, the laser beams were collimated and focused (100x oil immersion objective, NA 1.4, Olympus) onto the sample under an angle allowing for highly inclined thin illumination (Tokunaga et al., 2008). Fluorescence emission was filtered by a dichroic mirror and filter (ZT405/488/561rpc & ZET405/488/561NF, Chroma). PAmCherry emission was projected onto an EMCCD camera (iXon Ultra, 512x512 pixels, Andor). The pixel size was 96 nm. Transmission illumination was provided by an LED source and condenser (ASI Imaging and Olympus). Sample position and focus were controlled with a motorized piezo stage, a z-motor objective mount, and autofocus system (MS-2000, PZ-2000FT, CRISP, ASI Imaging). Movies of 20,000 frames at 20°C were acquired under continuous 561 nm laser excitation at 250 W/cm^2^ with an exposure times of 15 ms, or where indicated at 750 W/cm^2^ with an exposure time of 5 ms. Camera readout was 0.48 ms giving frame intervals of 15.48 ms or 5.48 ms, respectively. We also recorded a transmitted light snapshot for segmenting cells in each movie. For imaging SytoGreen, snapshots with 488 nm excitation with a 50 ms exposure time were acquired prior to PAmCherry imaging.

#### Localization and tracking

Single-molecule-tracking analysis was performed using custom-written MATLAB software (MathWorks) as previously described (Uphoff et al., 2014): fluorophore images were identified for localization by band-pass filtering and applying an intensity threshold to each frame of the movie. Candidate positions were used as initial guesses in a two-dimensional elliptical Gaussian fit for high-precision localization. Free fit parameters were x-position, y-position, x-width, y-width, elliptical rotation angle, intensity, background. Localizations were segmented based on cell outlines obtained from MicrobeTracker applied to the brightfield snapshots. Single-particle tracking analysis was performed by adapting the MATLAB implementation of the algorithm described in Crocker and Grier (1996). Positions were linked to a track if they appeared in consecutive frames within a window of 5 pixels (0.48 μm). When multiple localizations fell within the tracking window, tracks were linked such that the sum of step distances was minimized. We used a ‘memory’ parameter of 1 frame to allow for transient disappearance of the fluorophore within a track due to blinking or missed localization.

#### Measuring the diffusion of tracked molecules

We determined the mobility of each molecule by calculating an individual apparent diffusion coefficient, $D_{i}^{*}$, from the one-step mean-squared displacement (MSD) of the track using:

$$D_{i}^{*}=\frac{1}{4n\Delta t\;}\sum_{i=1}^{n}{\left[x\left(i\Delta t\right)-x\left(i\Delta t+\Delta t\right)\right]}^{2\;}+{\left[y\left(i\Delta t\right)-y\left(i\Delta t+\Delta t\right)\right]}^{2\;}$$

Where x(t) and y(t) are the coordinates of the molecule at time t, the frame time of the camera is Δt, and n is the number of frames over which the molecule is tracked. For a molecule diffusing with an apparent diffusion coefficient D^∗^, the probability of measuring a $D_{i}^{*}$ by tracking it overn frames, is given by Vrljic et al. (2002):

$$p\left(D_{i}^{*}\right)=\frac{1}{\left(n-1\right)!}*{\left(\frac{n}{D}\right)}^{n}*{\left(D_{i}^{*}\right)}^{n-1}*exp\left(\frac{-nD_{i}^{*}}{D}\right)$$

In order to determine the apparent diffusion coefficient, D^∗^, from the population of individual single-molecule $D_{i}^{*}$ values, longer tracks were truncated after 5^th^ localization (i.e., n=4). The $D_{i}^{*}$ distribution was then fitted with the equation for n=4:

$$p\left(D_{i}^{*}\right)=\frac{1}{6}*{\left(\frac{4}{D}\right)}^{4}*{\left(D_{i}^{*}\right)}^{3}*exp\left(\frac{-4D_{i}^{*}}{D}\right)$$

Fits were performed using maximum likelihood estimation in MATLAB. For unperturbed cells the protein diffusion distributions were fit with a model containing two molecular species with diffusion coefficients $D_{1}^{*}$ and $D_{2}^{*}$: representing immobile molecules bound to DNA for the entire trajectory, and mobile molecules diffusing and binding only transiently to DNA:

$$p\left(D_{i}^{*}\right)=\left[\frac{A_{1}}{6}*{\left(\frac{4}{D_{1}^{*}}\right)}^{4}*{\left(D_{i}^{*}\right)}^{3}*exp\left(\frac{-4D_{i}^{*}}{D_{1}^{*}}\right)\right]+\left[\frac{\left(1-A\right)}{6}*{\left(\frac{4}{D_{2}^{*}}\right)}^{4}*{\left(D_{i}^{*}\right)}^{3}*exp\left(\frac{-4D_{i}^{*}}{D_{2}^{*}}\right)\right]$$

where Aand 1−Aare the fraction of molecules found in each state. The localization uncertainty, σ_loc_, manifests itself as a positive offset of σ_loc_^2^/Δt in the D^∗^ value (Michalet and Berglund, 2012). Based on the estimated localization uncertainty of ∼35 nm for our measurements, we expected a positive shift in the mean D^∗^ value of immobile molecules to ∼0.7 μm^2^s^-1^. Where indicated error bars represent 95% confidence intervals obtained from fitting the D^∗^ distribution for 1000 bootstrap resamplings with replacement of individual segmented cells. For each bootstrap the tracks within the sampled cells were pooled and fitted as described above. To plot maps of tracks from mobile and immobile molecules, we used a threshold of 0.15 μm^2^s^-1^ to separate the populations.

#### Monte Carlo diffusion simulations

The apparent diffusion coefficients determined experimentally through particle tracking do not take into account three-dimensional confinement in the bacterial cell. We followed a similar rationale as before to remove this bias (English et al., 2011; Uphoff et al., 2013): we simulated Brownian motion confined within 3D cell volumes obtained from the segmented 2D brightfield images. The distance from the midline to the cell edge was used as the radius of a cylindrical volume for each length segment of a cell. For each cell diffusion simulations of the same number of molecules as measured experimentally were performed. Each 15 ms frame was split into 100 sub-frames with Gaussian-distributed displacements in each sub-frame. Each molecule trajectory was given a random starting time to mimic stochastic photoactivation, and a duration sampled from an exponential distribution with a mean time equal to our experimentally determined photobleaching lifetime (85 ms). The sub-frame distributions were then averaged to give a position for each frame, and a localization error sampled from a Gaussian distribution with σ_loc_ = 35 nm was added. The list of simulated localizations, with their corresponding frame numbers was then analyzed using the same algorithms with the same settings as for experimental data. The best estimate for the unbiased diffusion coefficient was determined by running the simulations for different D values between 0 and 10 μm^2^s^-1^ and selecting inputted D value from the simulated D^∗^ distribution which best approximates (based on the least-squares error) the experimentally obtained D^∗^ distribution. Since diffusion coefficients in DNA-free cells were much higher than in unperturbed cells we also performed experiments and simulations for Pol1 and MukB at 5.48 ms exposure times to verify the same underlying unbiased diffusion coefficients were obtained independent of the data acquisition conditions. 95% confidence intervals were estimated by fitting the experimental D^∗^ distribution for 1000 bootstrap resamplings with replacement of individual segmented cells as described previously. Simulations were then performed to determine inputted D value which best approximates the higher and lower confidence bounds from the experimentally determined D^∗^ values.

We hypothesized that the observed diffusion of DNA-binding proteins in unperturbed cells represented mobile molecules interconverting between D_free_ and D_bound_ states. By comparing diffusion in unperturbed and DNA-free cells, it is possible to estimate the relative occupation of the states but not the absolute duration a molecule spends in each state. To simulate molecules interconverting between these states, we used the D_free_ value based on the simulations of DNA-free cells, and a D_bound_ value of 0.04 μm^2^s^-1^. Because the D_mobile_ population appears as a single species the interconversions must occur on a timescale below the observation window per track (75 ms, 5 frames of 15 ms). We therefore simulated the duration of D_bound_, t_bound_, by randomly sampling from an exponential distribution with a mean of 1 ms. We performed simulations for a range of ratios of durations in the D_free_ and D_bound_ states by varying, t_free_, the duration of free diffusion between binding events. Using least-squares optimization, we determined the ratio which best recapitulated the experimental D^∗^_mobile_ value determined from fitting the experimental D^∗^ distribution.

#### Chromosome occupancy calculations

To estimate the percentage of the chromosome occupied by proteins we used literature estimates of the DNA footprint of each protein. RNAP (70bp; Ross and Gourse, 2005); HU (36bp; Gruber, 2014); H-NS (30bp; van der Valk et al., 2017); DNA gyrase (100bp; Reece and Maxwell, 1991). Where no DNA footprint estimates could be found we assumed a footprint of 10bp. The total bp occupied was calculated by the molecules/cell multiplied by the total fraction binding (including stable binding and transient binding) in Table 1, and the DNA footprint, giving 1.96Mb of DNA. Under the minimal media growth conditions in this study there are on average 1.5 chromosomes per cell, totaling 6.9Mb of DNA (Wang et al., 2005).

### Quantification and statistical analysis

Statistical details of experiments can be found in the figure legends. This includes exact value of samples, number of experiments and definition of dispersion measures (SD or SEM) between experiments. Microscopy images were randomly collected to obtain sufficient number of cells for each dataset. No data was excluded besides the specific criteria defined in the figure legends. Independent experiments were used to define the reproducibility of results.

## Supplemental information

Document S1. Figures S1–S6 and Tables S1 and S2

Document S2. Article plus supplemental information
