# Supplementary material for: Transient non-specific DNA binding dominates the target search of bacterial DNA-binding proteins
Source: Mol Cell. 2021 Apr 1;81(7):1499–1514.e6. doi: 10.1016/j.molcel.2021.01.039 (PMC8022225; doi:10.1016/j.molcel.2021.01.039)
Supplement: Document S1. Figures S1–S6 and Tables S1 and S2 [file mmc1.pdf]

Molecular Cell, Volume *81*

Supplemental information

Transient non-specific DNA binding dominates the target search of bacterial DNA-binding proteins

Mathew Stracy, Jakob Schweizer, David J. Sherratt, Achillefs N. Kapanidis, Stephan Uphoff, and Christian Lesterlin

SUPPLEMENTAL ITEMS:

Figure S1: DNA-binding proteins diffusion analysis. Related to Fig. 1 and 2.

Figure S2: Quantification of DNA degradation efficiency. Related to Fig. 3.

Figure S3: Min oscillation period analysis. Related to Fig. 3E.

Figure S4: Effects of chromosome degradation on protein production and diffusion. Related to Fig. 3.

Figure S5: Identification of DNA-free cells. Related to Fig. 3 and 4.

Figure S6: Influence of exposure time. Related to Fig. 6.

Table S1: Functionality of the tested fusion proteins. Related to Fig. 1 and Table 1.

Table S2: Strains and plasmids used in this study. Related to the STAR methods section.

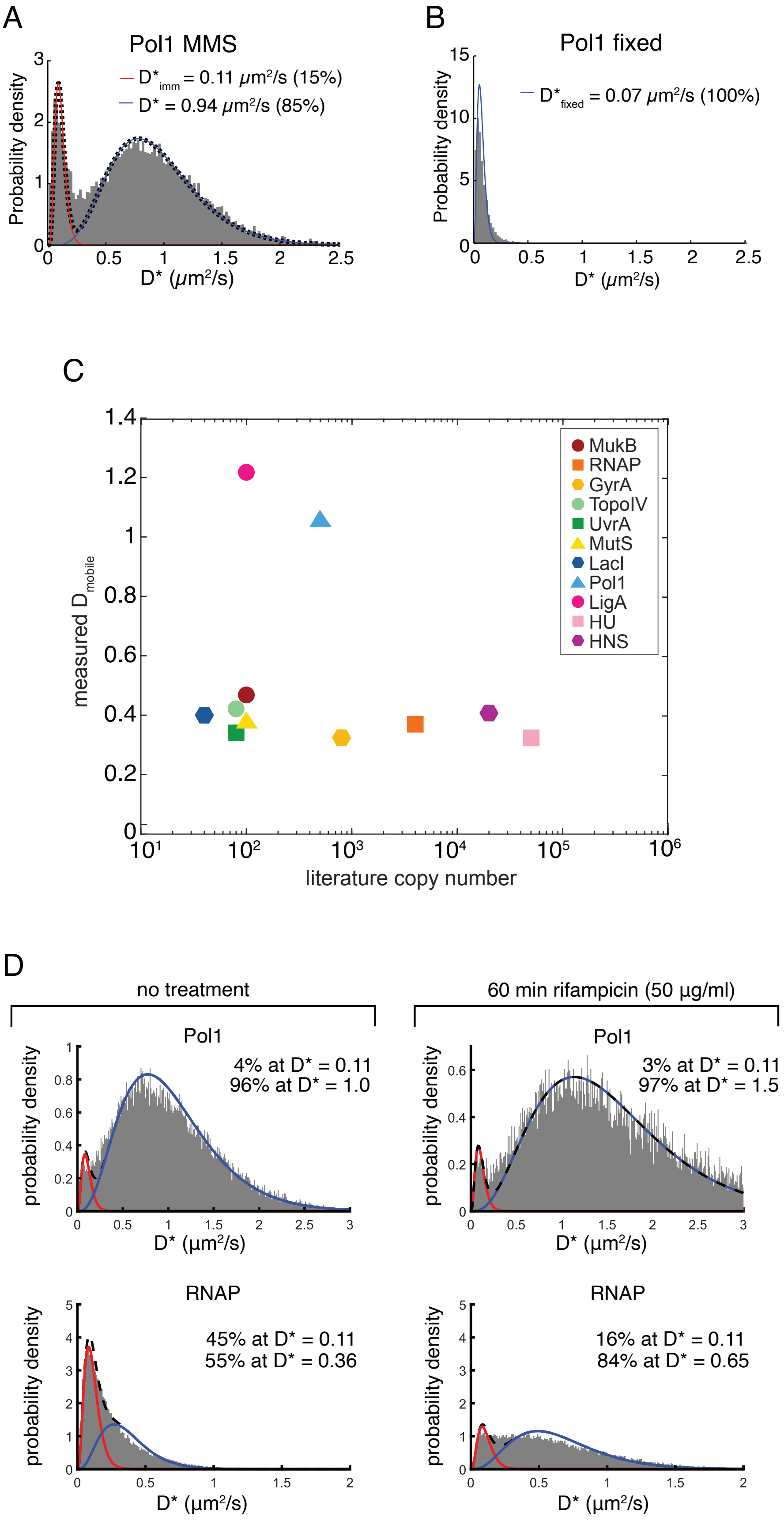

A
Pol1 MMS
D*imm = 0.11 μm²/s (15%)
D* = 0.94 μm²/s (85%)
Probability density
D* (μm²/s)
B
Pol1 fixed
D*fixed = 0.07 μm²/s (100%)
Probability density
D* (μm²/s)
C
measured Dmobile
literature copy number
MukB
RNAP
GyrA
TopoIV
UvrA
MutS
LacI
Pol1
LigA
HU
HNS
D
no treatment
60 min rifampicin (50 μg/ml)
Pol1
4% at D* = 0.11
96% at D* = 1.0
Pol1
3% at D* = 0.11
97% at D* = 1.5
RNAP
45% at D* = 0.11
55% at D* = 0.36
RNAP
16% at D* = 0.11
84% at D* = 0.65
probability density
D* (μm²/s)

Figure S1

Figure S1: DNA-binding proteins diffusion analysis. Related to Fig. 1 and 2. (A) D* histograms and model fit of PolA-PAmCherry in cells treated with methyl methanesulfonate (MMS). (B) D* histograms of PolA-PAmCherry in fixed cells, fitted with a model of immobile molecules only. (C) Scatter plot of experimentally determined D_{mobile} diffusion of 12 DNA-binding proteins against their intracellular copy number. The fitted D_{mobile} value extracted from a 2 species fit to the histograms of apparent diffusion coefficients, D*, presented in Figure 1. The copy number estimates are from literature sources are presented in Table 1. (D) Distributions histograms of apparent diffusion, D*, of three DNA-binding proteins: DNA polymerase 1 (Pol1) and RNA polymerase (RNAP) before (left) and after 60 mins treatment with 50 µg/ml rifampicin (right). Distributions are fitted with a 2-species model of immobile (in red) and mobile molecules (in blue).

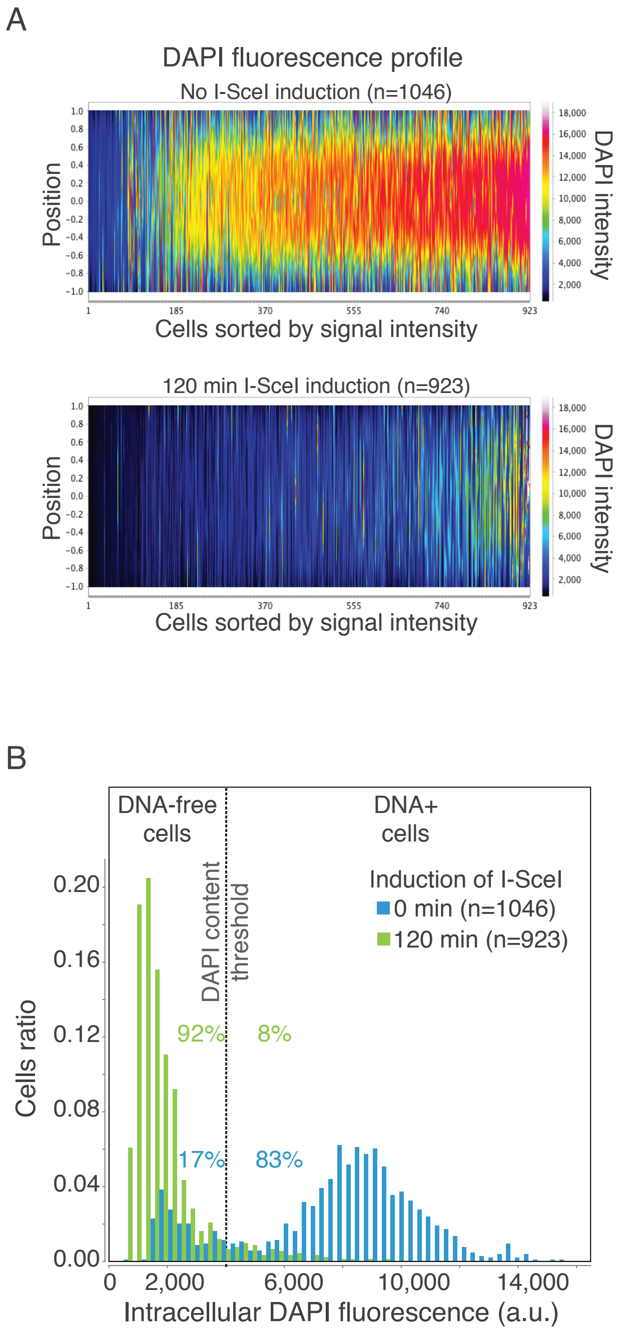
A
DAPI fluorescence profile
No I-SceI induction (n=1046)
Position
Cells sorted by signal intensity
DAPI intensity
120 min I-SceI induction (n=923)
Position
Cells sorted by signal intensity
DAPI intensity
B
DNA-free cells
DNA+ cells
DAPI content threshold
Induction of I-SceI
0 min (n=1046)
120 min (n=923)
92%
8%
17%
83%
Cells ratio
Intracellular DAPI fluorescence (a.u.)

Fig. S2

Figure S2: Quantification of DNA degradation efficiency. Related to Fig. 3. (A) Fluorescence profiles show the distribution and intensity of DAPI signal in individual cells normalized by the cell length and sorted from left to right by increasing mean intensity. Fluorescence profile of OT / pSN1(pP_{BAD}-*I-SceI*) strain before (Top panel) and 120 min after I-SceI induction (lower panel) are shown. (B) Histograms of DAPI-stained DNA intracellular fluorescence in OT / pSN1(PBADI-SceI) cell population, before (blue bars) and 120 min after I-SceI induction (green bars). The percentages of cells below and above a DAPI content threshold (grey dash line) are shown. Before I-SceI induction, 17 % of cells already exhibit DNA loss likely due to the leakiness I-SceI expression from PBAD promoter. 120 min after arabinose-induced I-SceI expression, this proportion increases to 92 % of the population, with 8 % of cells still containing DNA (n= cells analyzed).

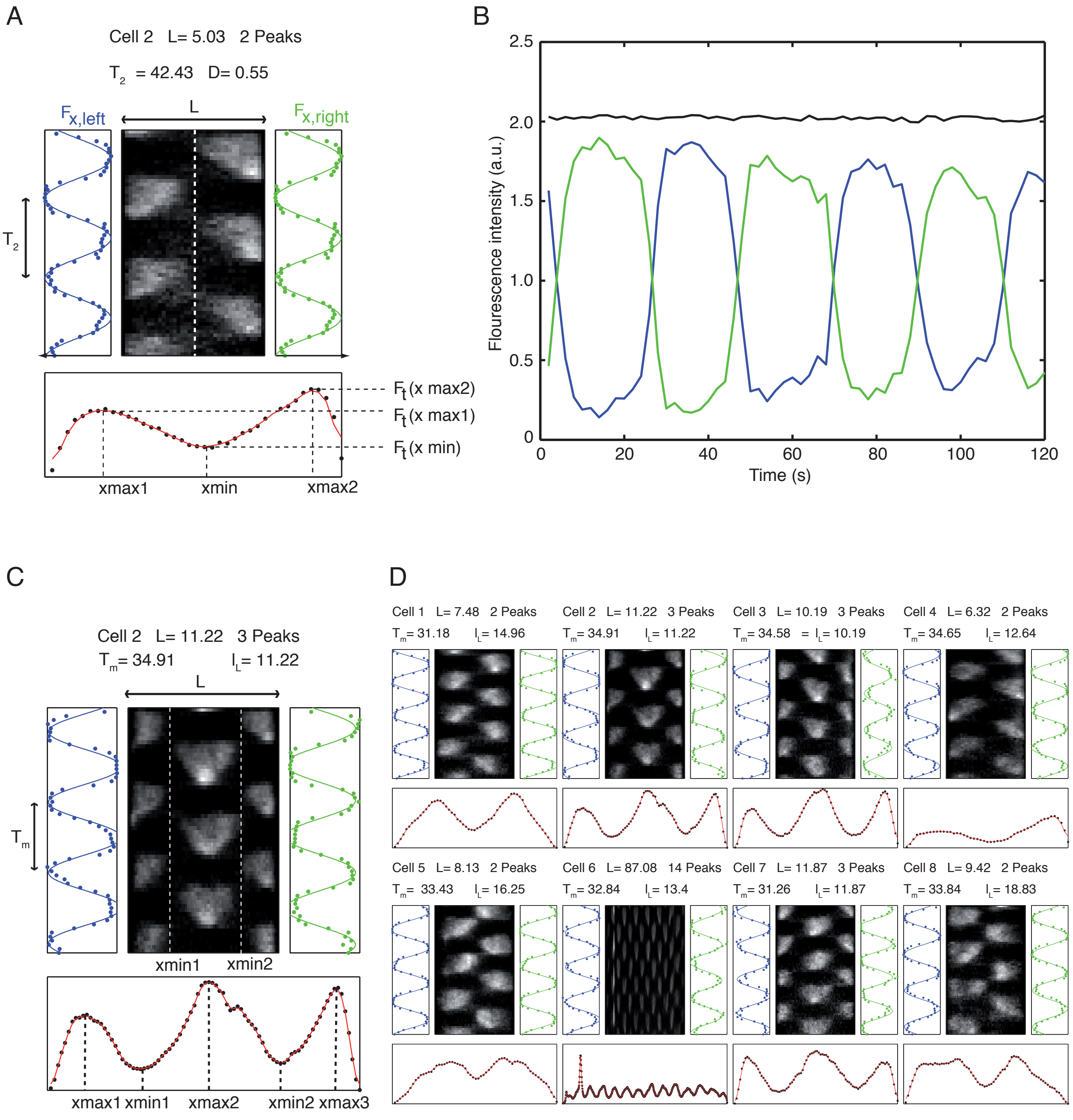
A
Cell 2 L= 5.03 2 Peaks
T2 = 42.43 D= 0.55
Fx,left
L
Fx,right
T2
Ft(x max2)
Ft(x max1)
Ft(x min)
xmax1
xmin
xmax2
B
Flourescence intensity (a.u.)
Time (s)
C
Cell 2 L= 11.22 3 Peaks
Tm= 34.91 lL= 11.22
L
Tm
xmin1
xmin2
xmax1 xmin1 xmax2 xmin2 xmax3
D
Cell 1 L= 7.48 2 Peaks
Tm= 31.18 lL= 14.96
Cell 2 L= 11.22 3 Peaks
Tm= 34.91 lL= 11.22
Cell 3 L= 10.19 3 Peaks
Tm= 34.58 = lL= 10.19
Cell 4 L= 6.32 2 Peaks
Tm= 34.65 lL= 12.64
Cell 5 L= 8.13 2 Peaks
Tm= 33.43 lL= 16.25
Cell 6 L= 87.08 14 Peaks
Tm= 32.84 lL= 13.4
Cell 7 L= 11.87 3 Peaks
Tm= 31.26 lL= 11.87
Cell 8 L= 9.42 2 Peaks
Tm= 33.84 lL= 18.83

Fig. S3

Figure S3: Min oscillation period analysis. Related to Fig. 3E. (A) Kymograph and concentration profiles of the fluorescence signal of MinC protein in exponentially growing *E. coli* cells. The width of the kymograph corresponds to the length L of the cell. Upon vertical splitting of the kymograph (white dashed line) and integration over space (x), the time-dependent intensity signals Fx,left(t) and Fx,right(t) are obtained. The oscillation period T2 can be calculated by periodic fitting (blue and green lines on the left and right of the kymograph). Upon integration over time (t), a spatial concentration profile of the MinC proteins is obtained (black data points below the kymograph) and fitted (red curve). The depth D of the profile is calculated from the heights of the maxima and the minimum. (B) Oscillation of fluorescence over time in cell halves. When the time-dependent fluorescence is integrated, an almost perfect constant line is obtained (black curve). This is due to the normalization of the intensity of each pixel in respect to the total fluorescence signal at each time point. In consequence, the two periodic curves for the left and right kymograph halves are perfectly symmetric (blue and green curve). (C) Kymograph and concentration profiles of the fluorescence signal of MinC protein in filamentous cells. In filamentous cells, the time-averaged concentration profile can show more than two peaks. The distance between two peaks corresponds to half an intrinsic wavelength of the Min system. For the calculation of the oscillation period Tm in filamentous cells, the kymograph is split along the position of the minima xmin (white dashed lines in the kymograph) resulting in several stripes with a periodic pattern. Integration along x provides a periodic functions Fx,i(t) from which oscillation period Ti can be calculated. Tm represents the average for these multiple oscillation periods. (D) Kymographs of different filamentous cells show different number of oscillations. Most of them show only a single (cells 1, 4, 5 and 8) or double oscillation (cells 2, 3 and 7). In the given example one cells shows 14 peaks, which corresponds to 13 oscillations and thus 6.5 wavelengths. The respective wavelength can be then calculated from the length of the cell and the number of oscillations.

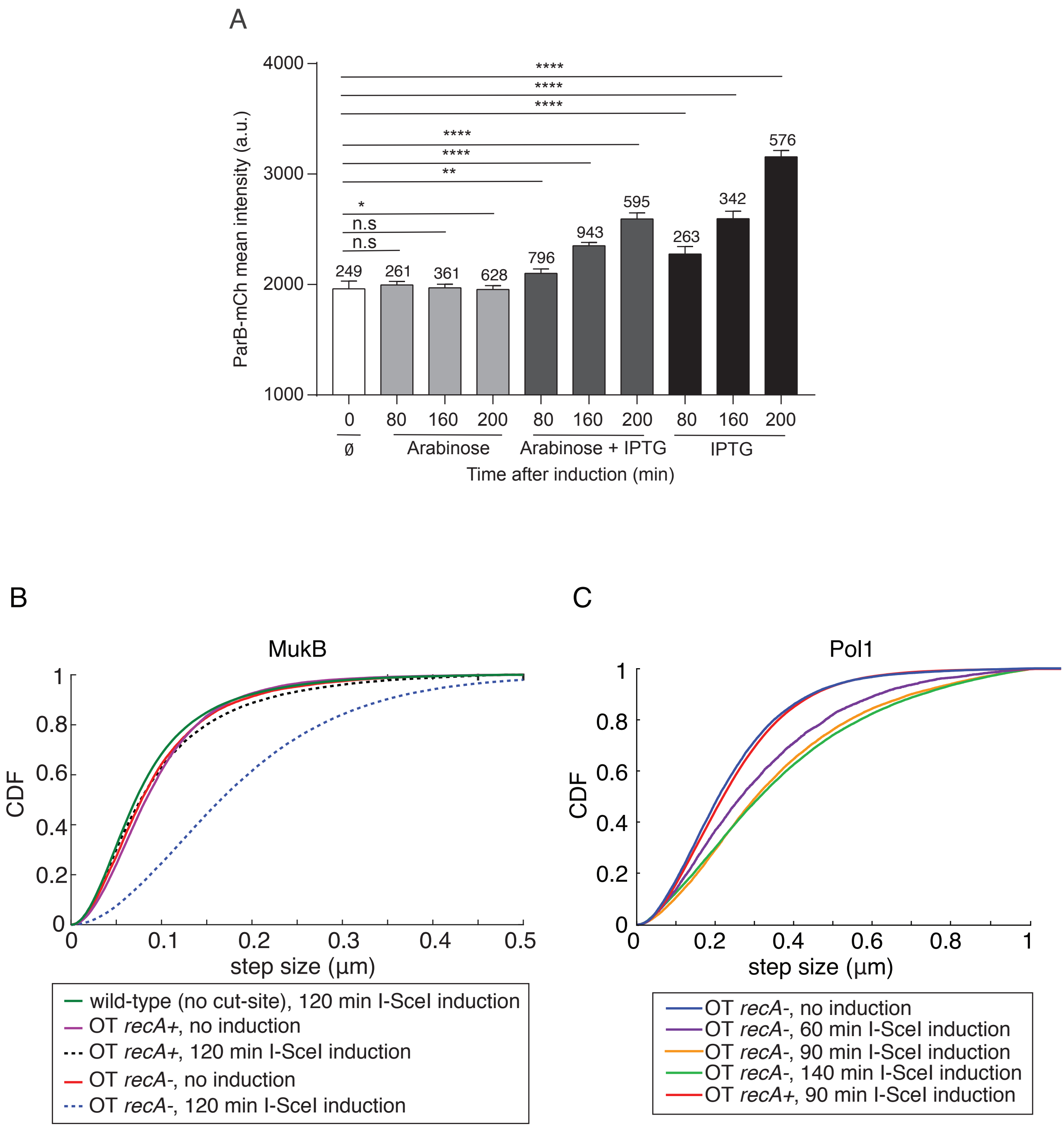
A
ParB-mCh mean intensity (a.u.)
4000
3000
2000
1000
****
****
****
****
****
**
*
n.s
n.s
249
261
361
628
796
943
595
263
342
576
0
80
160
200
80
160
200
80
160
200
Ø
Arabinose
Arabinose + IPTG
IPTG
Time after induction (min)
B
MukB
CDF
step size (µm)
wild-type (no cut-site), 120 min I-SceI induction
OT recA+, no induction
OT recA+, 120 min I-SceI induction
OT recA-, no induction
OT recA-, 120 min I-SceI induction
C
Pol1
CDF
step size (µm)
OT recA-, no induction
OT recA-, 60 min I-SceI induction
OT recA-, 90 min I-SceI induction
OT recA-, 140 min I-SceI induction
OT recA+, 90 min I-SceI induction

Fig. S4

Figure S4: Effects of chromosome degradation on protein production and diffusion. Related to Fig. 3. (A) Maintenance of protein synthesis activity upon induction of DNA degradation. Quantification of ParB-mCherry intracellular signal (a.u., arbitrary unit) produced from IPTG inducible pSN70 plasmid (P_{lac} *I-SceI*) in OT strain. I-SceI expression from pSN1 plasmid is induced by arabinose 0.2 %. Chromosome degradation alone has little impact on ParB-mCherry production. Over the course of the experiment (200 minutes), ParB-mCherry production is induced by IPTG with or without chromosome degradation. Error bars indicate the standard error and n = the numbers of cells analyzed. Two tailed P-values from Mann-Whitney non-parametric test are indicated by (n.s) non-significant P-value > 0.05, * for P-value < 0.05, ** for P-value < 0.01 and **** for P-value < 0.001. (B) Cumulative distribution plot of tracked MukB-PAmCherry trajectory step size in cells before and 120 mins after I-Scel induction. Protein diffusion remains unchanged *recA* proficient cells (*recA+*) before (magenta line) after induction (black dashed line), whereas diffusion increases after induction in *recA*- cells (blue dashed line). (C) Cumulative distribution plot of tracked Pol1-PAmCherry trajectory step size in (recA-) cells before and after I-Scel induction. Protein diffusion increases with I-Scel induction time from 0 to 90 mins, but increases only modestly beyond 90 mins induction.

A

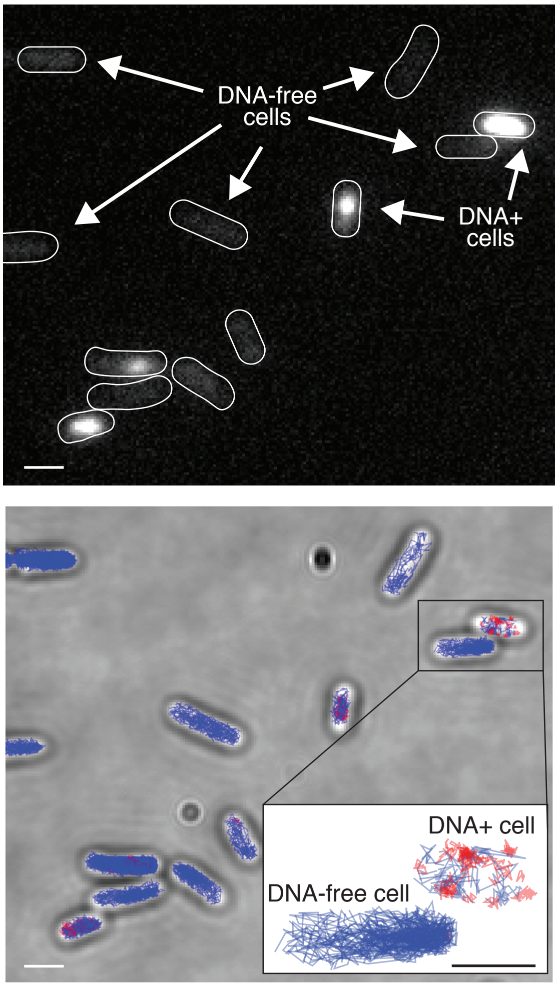
DNA-free
cells
DNA+
cells
DNA+ cell
DNA-free cell

B

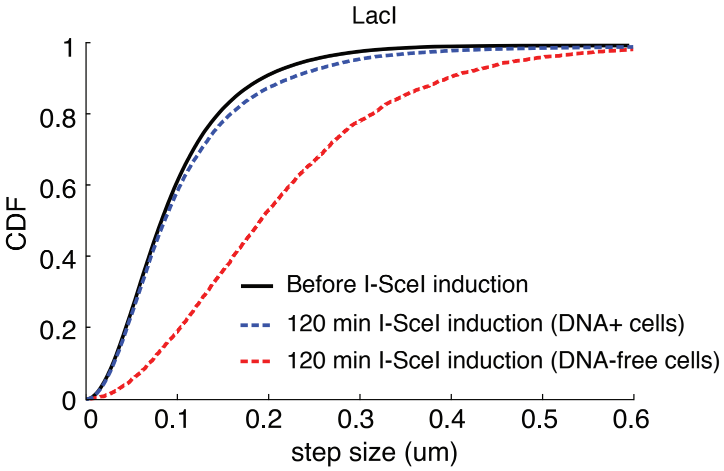
LacI
CDF
1
0.8
0.6
0.4
0.2
0
0
0.1
0.2
0.3
0.4
0.5
0.6
step size (um)
Before I-SceI induction
120 min I-SceI induction (DNA+ cells)
120 min I-SceI induction (DNA-free cells)

Fig. S5

Figure S5: Identification of DNA-free cells. Related to Fig. 3 and 4. A fraction of cells did not undergo full DNA degradation and these cells showed little change in the diffusion profiles. (A) Fluorescence image of SytoGreen stained DNA in cells after 120 mins of I-SceI induction showing DNA+ and DNA-free cells (top). The brightfield image of the same cells overlaid with the categorized trajectories of RNAP-PAmCherry tracks with immobile molecules in red and mobile molecules in blue (bottom). The lower-right insert presents a zoom of one DNA+ and one DNA-free cell. (B) Cumulative distribution of LacI-PAmCherry trajectory displacement steps in cells having high (DNA+ cells in blue) or low (DNA-free cells in red) SytoGreen fluorescence compared to the unperturbed cells (in black).

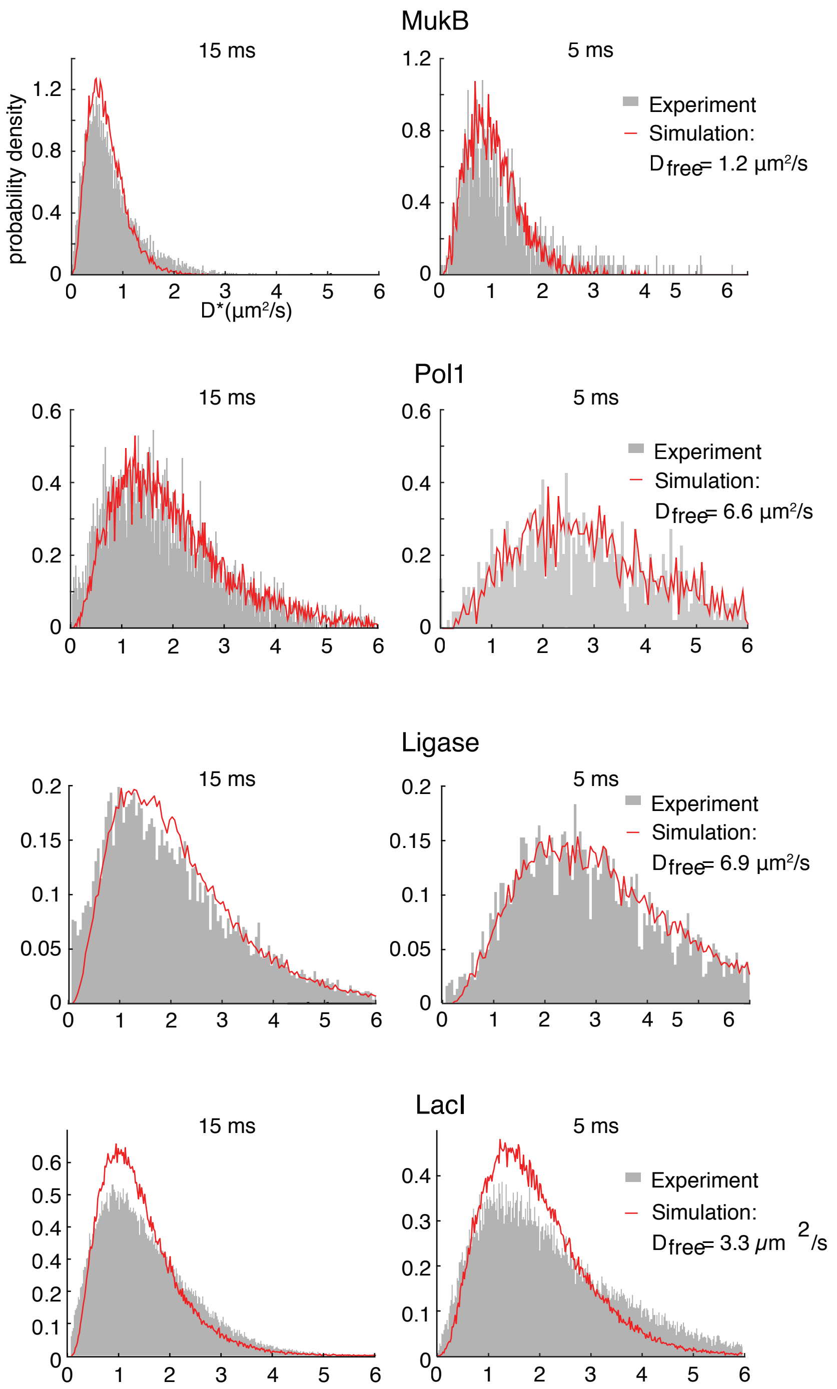

MukB
15 ms
5 ms
probability density
D*(µm²/s)
Experiment
Simulation:
Dfree= 1.2 µm²/s
Pol1
Dfree= 6.6 µm²/s
Ligase
Dfree= 6.9 µm²/s
LacI
Dfree= 3.3 µm²/s

Fig. S6

Figure S6: Influence of exposure time. Related to Fig. 6. D* distribution (grey bars) of MukB-PAmCherry, Pol1-PAmCherry, LigA-PAmCherry and LacI-PAmCherry in DNA-free cells 120 min after I-SceI induction measured with an exposure time of 15 ms (left) and 5 ms (right). The D* distribution generated from simulated molecule trajectories with the same diffusion coefficient, D_{free}, at 15 ms (left) and 5 ms (right) exposure times.

Table S1. Functionality of the tested fusion proteins. Related to Fig. 1 and Table 1.

Fusion protein	Functionality tests	Reference
**RNA Polymerase (β' subunit)**	The fusion strain shows WT growth rate and cell size. The fusion strain shows expected decrease in DNA binding upon incubation with the transcription inhibiting antibiotic rifampicin.	Stracy et al., 2015 Endesfelder et al 2013
**DNA polymerase 1 (PolA)**	The fusion strain shows WT growth rate and cell size. The fusion strain shows WT levels of sensitivity to DNA methylation damage with methyl methanesulfonate (MMS). The fusion protein shows expected increase in DNA binding upon incubation with MMS.	Uphoff et al., 2013
**MukBEF (MukB subunit)**	The cells have a Muk+ phenotype with WT growth rate and cell size (Muk- cells are temperature sensitive and cannot grow at 37, and generate anucleate cells).	Badrinarayanan et al., 2012
**DNA Ligase (LigA)**	The fusion strain shows WT growth rate and cell size, and WT levels of sensitivity to DNA methylation damage with methyl methanesulfonate (MMS). The fusion protein shows expected increase in DNA binding upon incubation with MMS.	Uphoff et al., 2013
**Lac repressor (LacI)**	The fusion strain shows WT growth rate and cell size. The fusion strain shows expected decrease in binding upon IPTG addition and in strains with the LacO operator sites removed.	Garza de Leon et al., 2017
**Heat Unstable protein (HU)**	The fusion strain shows WT growth rate and cell size. The *hupB-PAmcherry* fusion is similar to *hupB-mcherry* fusion used in Starcy et al., 2015 (same SSAGSAAGSGEF flexible linker).	Similar C-ter fusion to *hupB* such as that used in Starcy et al, 2015
**Histone-like nucleoid structuring protein (H-NS)**	The fusion strain shows WT growth rate and cell size. The *hns-PAmcherry* C-term fusion (with SSAGSAAGSGEF linker) is similar to *hns-mcherry* fusion used in Gao et al., 2017 (with GSAGSAAGSGEF linker).	Similar C-ter fusion to *hns as used in* Gao et al., 2017
**MutS**	The fusion strain shows WT growth rate and cell size, and has the same mutation rate as WT strain. The fusion protein shows expected increase in DNA binding upon incubation with MMS mutagen.	Uphoff et al., 2016
**Topoisomerase IV (ParC subunit)**	The fusion strain shows WT growth rate and cell size, with no defect in chromosome segregation as assessed by flow cytometry. The fusion protein shows expected increase in DNA binding following incubation with the type II topoisomerase inhibitor norfloxacin.	Zawadzki et al., 2015
**UvrA**	The fusion strain shows WT growth rate, cell size and WT levels of sensitivity to UV damage. The fusion protein shows expected increase in DNA binding following exposure to UV.	Stracy et al., 2016
**DNA gyrase (GyrA subunit)**	The fusion strain shows WT growth rate and cell size. The purified fusion protein can relax supercoiled DNA in vitro. The fusion protein shows expected increase in DNA binding following incubation with the type II topoisomerase inhibitor norfloxacin.	Stracy et al., 2019

Table S2. Strains and plasmids used in this study. Related to the STAR Methods section.

Strain	Relevant Genotype	Source or Reference
**MG1655**	F- lambda- *ilvG- rfb*-50 *rph*-1	CGSC#: 7740
**TB28**	MG1655 *ΔlacIZYA*	Bernhardt and de Boer, 2005
TB28 I-SceI^{CS}-ilvA	TB28 *I-SceI^{CS}-ilvA-FRT* (3953 kb)	TB28 × P1.*I-SceI^{CS}-ilvA* to Cm^{r}, *cat* removed via pCP20
**TB28 *I-SceI^{CS}-ydeO***	TB28 *I-SceI^{CS}-ydeO-FRT-cat-FRT* (1580 kb)	TB28 × P1. *I-SceI^{CS}-ydeO* to Cm^{r,}
**OT**	TB28 *I-SceI^{CS}-ilvA-FRT, I-SceI^{CS}-ydeO-FRT*	TB28 *I-SceI^{CS}-ilvA* × P1.*I-SceI^{CS}-ydeO* to Cm^{r}, *cat* removed via pCP20
**RNAP-PAmCherry**	MG1655 *rpoC-PAmCherry-FRT-kan-FRT*	Stracy et al., 2015
**HU-PAmCherry**	MG1655 *hupB-PAmCherry-FRT-kan-FRT*	Stracy et al., 2015
**HN-S-PAmCherry**	MG1655 *Hns-PAmCherry-FRT-kan-FRT*	Stracy et al., 2015
**FIS-PAmCherry**	MG1655 *fis-PAmCherry-FRT-kan-FRT*	Uphoff et al., 2013
**LacI-mCherry**	MG1655 *LacI-PAmCherry*	Garza de Leon et al., 2017
**Pol1-PAmCherry**	MG1655 *polA-PAmCherry-FRT-kan-FRT*	Uphoff et al., 2013
**LigA-PAmCherry**	MG1655 *ligA-PAmCherry-FRT-kan-FRT*	Uphoff et al., 2013
**UvrA-PAmCherry**	MG1655 *uvrA-PAmCherry-FRT-kan-FRT*	Stracy et al., 2016
**MutS-PAmCherry**	MG1655 *mutS-PAmCherry-FRT-kan-FRT*	Uphoff et al., 2016
**TopoIV-PAmCherry**	MG1655 *parC-PAmCherry-FRT-kan-FRT*	Zawadzki et al., 2015
**MukB-PAmCherry**	MG1655 *mukB-PAmCherry-FRT-kan-FRT*	Badrinarayanan et al., 2012
**GyrA-PAmCherry**	MG1655 *gyrA-PAmCherry-FRT-kan-FRT*	Stracy et al., 2019
recA- strain	TB28 *recAT233C-Tet*	Lesterlin et al., 2014
**MinC-Ypet**	*minC-Ypet*	Bisicchia et al., 2013
**OT RNAP-PAmCherry**	OT *rpoC-PAmCherry-FRT-kan-FRT*	OT x P1. RNAP-PAmCherry to Km^{r}
**OT Pol1-PAmCherry**	OT *polA-PAmCherry-FRT-kan-FRT*	OT x P1. Pol1-PAmCherry to Km^{r}
**OT LigA-PAmCherry**	OT *ligA-PAmCherry-FRT-kan-FRT*	OT x P1. LigA-PAmCherry to Km^{r}
**OT MukB-PAmCherry**	OT *mukB-PAmCherry-FRT-kan-FRT*	OT x P1. MukB-PAmCherry to Km^{r}
**OT LacI-PAmCherry**	OT */ p lacI-PAmCherry*	Transformation of p *lacI-PAmCherry* into OT strain
**OT LacI^{41}-PAmCherry**	OT */ p lacI^{41}-PAmCherry*	Transformation of p *lacI^{41}-PAmCherry* into OT strain
**OT Free PAmCherry**	OT pBAD\HisB PAmCherry1	Transformation of pBAD\HisB PAmCherry1into OT strain
**OT FIS-PAmCherry**	OT *fis-PAmCherry-FRT-kan-FRT*	OT x P1. FIS-PAmCherry to Km^{r}
**OT *recA-***	OT *recAT233C-Tet*	OT x P1. *recAT233C-Tet* to Tc
**OT RNAP-PAmCherry *recA-***	OT *rpoC-PAmCherry-FRT-kan-FRT*	OT RNAP-PAmCherry x P1. *recAT233C-Tet* to Tc
**OT Pol1-PAmCherry *recA-***	OT *polA-PAmCherry-FRT-kan-FRT*	OT Pol1-PAmCherry x P1. *recAT233C-Tet* to Tc
**OT LigA-PAmCherry *recA-***	OT *ligA-PAmCherry-FRT-kan-FRT*	OT LigA-PAmCherry x P1. *recAT233C-Tet* to Tc

**OT MukB-PAmCherry *recA-***	OT *mukB-PAmCherry-FRT-kan-FRT*	OT MukB-PAmCherry x P1. *recAT233C-Tet* to Tc to Km^r
**OT LacI-PAmCherry *recA-***	OT */ P lacI-PAmCherry*	Transformation of *P lacI-PAmCherry* into OT *recA-*
**OT LacI^{41}-PAmCherry *recA-***	OT */ P lacI^{41}-PAmCherry*	Transformation of *P lacI^{41}-PAmCherry* into OT *recA-*
**OT Free PA-mCherry *recA-***	OT pBAD\HisB PAmCherry1	Transformation of pBAD\HisB PAmCherry1 into OT *recA-* strain
**Plasmids**		
**p *lacI-PAmCherry***	LacI-PAmCherry producing plasmid	Garza de Leon et al., 2017
**p *lacI^{41}-PAmCherry***	LacI^{41}-PAmCherry producing plasmid	Garza de Leon et al., 2017
**pBAD\HisB PAmCherry1**	PAmCherry1 producing plasmid	Endesfelder et al., 2013
**pCP20**	Flp expression plasmid	Datsenko et al., 2000
**p*ParBmCherry* (pSN70)**	IPTG inducible expression of N-terminal fusion mCherry-$ParB_{PMT1}$	Nolivos et al., 2019
**pI-SceI (pSN1)**	Arabinose inducible expression of I-SceI endonuclease	Gift from Sophie Nolivos
